# Using genome-wide data to ascertain taxonomic status and assess population genetic structure for Houston toads (*Bufo* [= *Anaxyrus*] *houstonensis*)

**DOI:** 10.1038/s41598-024-53705-w

**Published:** 2024-02-08

**Authors:** Shashwat Sirsi, David Rodriguez, Michael R. J. Forstner

**Affiliations:** Department of Biology, Texas State University, 601 University Drive, San Marcos, TX 78666 USA

**Keywords:** Phylogenetics, ddRADseq, *Bufo* [= *Anaxyrus*] *houstonensis*, Endangered, Historic gene flow, Molecular ecology, Conservation biology, Population genetics

## Abstract

The Houston toad (*Bufo* [= *Anaxyrus*] *houstonensis*) is an endangered amphibian with a small geographic range. Land-use changes have primarily driven decline in *B. houstonensis* with population supplementation predominant among efforts to reduce its current extinction risk. However, there has been historic uncertainty regarding the evolutionary and conservation significance of *B. houstonensis*. To this end, we used 1170 genome-wide nuclear DNA markers to examine phylogenetic relationships between our focal taxon, representatives of the Nearctic *B. americanus* group, and *B. nebulifer*, a sympatric Middle American species. Phylogenetic analyses indicate *B. houstonensis* is a taxon that is distinct from *B. americanus*. We corroborated such genetic distinctiveness with an admixture analysis that provided support for recent reproductive isolation between *B. americanus* and *B. houstonensis*. However, ABBA-BABA tests for ancient admixture indicated historic gene flow between Nearctic species while no signal of historic gene flow was detected between Nearctic and Middle-American species. We used an admixture analysis to recognize four Management Units (MU) based on observed genetic differentiation within *B. houstonensis* and recommend captive propagation, population supplementation, and habitat restoration efforts specific to each MU. Our results re-affirm the evolutionary novelty of an endangered relict.

## Introduction

Amphibians are recognized as one of the most imperiled vertebrate groups, with nearly a third of all species threatened with extinction, and they also represent the largest proportion of data deficient species^1,2^. Various threats to amphibians have been documented (e.g., habitat loss, climate change, and infectious diseases), with synergies between extrinsic threats and amphibian ecological/life history traits resulting in higher extinction risks for large-bodied amphibians with small geographic ranges^3–5^.

**Figure 1:**
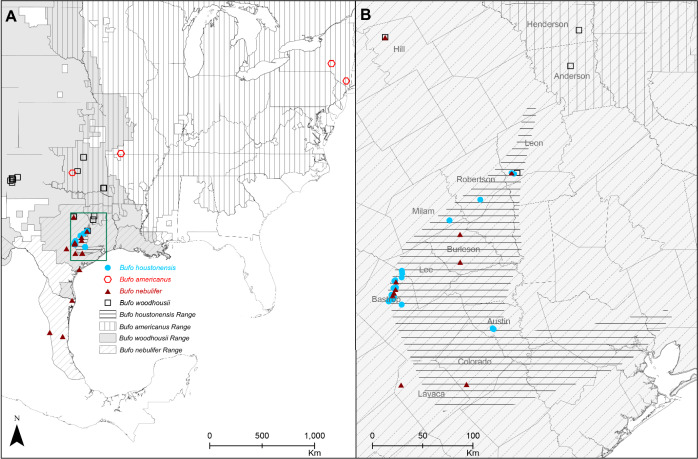
(**A**) Map of sampling localities relative to known distribution for 8 American Toads (*Bufo* [= *Anaxyrus*] *americanus*), 48 Houston toads (*B. houstonensis*), 18 Woodhouse’s Toads (*B. woodhousii*), and 19 Gulf Coast Toads (*B. nebulifer*). (**B**) The study included 48 samples of *B. houstonensis* from Bastrop, Lee, Austin, Leon, Milam, and Robertson counties within species range. © 2020 Texas Department of Transportation, U.S. Census, TomTom North America, Inc., U. S. Department of Agriculture, National Agricultural Statistics Service, and Global Biodiversity Information Facility (https://www.gbif.org/species/2422886; 2422872; 5217017; 2422404).

Houston toads (*Bufo* [= *Anaxyrus*] *houstonensis*) serve as an example of an amphibian taxon with a small geographic range and high risk of extinction^6–8^. Historical classification of North American toads based on morphological similarity placed *B. houstonensis* in the *Bufo americanus* species group along with *Bufo americanus*, *Bufo terrestris*, *Bufo hemiophrys*, *Bufo microscaphus*, *Bufo woodhousii*, *Bufo fowleri*, and *Bufo velatus*^9,10^. Additionally, *B. houstonensis* is considered a late Pleistocene relict. Populations of *B. houstonensis* were left behind in Texas after a widely distributed parental species (i.e., a precursor to present day American Toads [*B. americanus*]) experienced a large range expansion to the north, following retreat of the Wisconsin glaciation^9^. The contemporary range of *B. houstonensis* encompasses nine counties (i.e., Austin, Bastrop, Burleson, Colorado, Lavaca, Lee, Leon, Milam, and Robertson; Fig. 1) in Southeast and Central Texas^11^; however, it was historically present but extirpated from Brazos, Freestone, Fort Bend, Grimes, Harris, and Liberty counties. Land-use changes have predominantly driven their decline given that the toad is a habitat specialist with a strong preference for deep, sandy soils that are currently most often associated with forest cover^6,12–14^. Owing to continued population declines since *B. houstonensis* was first described^15^, it was designated as an endangered species on national and international lists^7,8^.

Despite long-term, broad-scale declines and being the first amphibian species to be listed as Federally Endangered, there have been few attempts to characterize genetic variation within *B. houstonensis.* McHenry’s^16^ range-wide study of population genetic structure in *B. houstonensis* recovered nine, largely geographically concordant clusters (i.e., five in Bastrop County and one each in Austin, Milam, Colorado, and Leon Counties) that did not exhibit reciprocal monophyly of mtDNA haplotypes but differed significantly in nuDNA allele frequencies. Further, both mtDNA sequences and microsatellite genotypes showed that *B. houstonensis* from Austin County were highly differentiated from all other *B. houstonensis*^16^. McHenry also reported low levels of contemporary genetic connectivity but considerable range-wide allelic diversity that may have been due to a larger historical species range and higher historical population connectivity. As a result, management recommendations were to, at minimum, supplement Austin and Bastrop populations while protecting and restoring dispersal corridors to enhance future population connectivity^16^.

Further, there has been historic uncertainty over the taxonomic status of *B. houstonensis* since these toads are only slightly differentiated in morphology and mating call from *B. americanus*^17–19^. Phylogenetic relationships within the *Bufo americanus* species group have focused on more widely distributed species while *B. houstonensis* has received considerably less attention^20–22^. Phylogenetic analyses that included *B. houstonensis* corroborated its placement within the *B. americanus* group but did not resolve relationships between *B. houstonensis* and *B. americanus*^16,23,24^. These previous analyses were based on mtDNA. Variation in nuclear and mitochondrial genes may be strikingly different for a single species for a variety of reasons such as sex-biased dispersal, incomplete lineage sorting, and asymmetric introgression. The mode of transmission of mitochondrial (i.e., maternal) and nuclear (i.e., biparental) genes further explains differences in sensitivity of each marker to fluctuations in demography and spatial range^25^. Given such potential for mitonuclear discordance, we sought to resolve these taxonomic ambiguities using several independent markers. Information regarding the genetic distinctiveness of *B. houstonensis* is critical to establishing whether costly propagation and supplementation efforts continue to be warranted.

Here we present a high-resolution genomic analysis of *B. houstonensis*, two closely related toad species in the *B. americanus* group, and a sympatric Middle American toad species (*Bufo* [= *Incilius*] *nebulifer*) to primarily reconcile uncertainties in the taxonomic status of *B. houstonensis*. We applied a double digest restriction-site associated DNA sequencing (ddRADseq) approach for genome-wide sampling of several nuclear loci and reconstructed phylogenetic relationships using a matrix of concatenated loci. Additionally, we sought to use our increased density of marker sampling to consolidate McHenry’s^16^ assessment of intraspecific genetic variation in *B. houstonensis*.

## Results

### Phylogenetic relationships and divergence times among Nearctic and Middle American taxa

We obtained a total 11,563,297 raw reads with 124,337 ± 74,570 (Mean ± SD) reads per sample (Table S1). We observed a maximum individual pairwise data-missingness of 0.99 across clustering thresholds with Pearson’s coefficient of correlation between data-missingness and genetic distance ranging from 0.53 to 0.70. Specifically, we observed a very low proportion of shared loci between our Nearctic and Middle American species, with the level of such missing data independent of the clustering threshold used to establish homology among reads (Figs. S1–S12). We observed, on average, ~ 4% (*n* = 50) of all loci recovered in *B. nebulifer* samples were shared with Nearctic study species. Within our Nearctic study group, on average, ~ 60% (*n* = 729) of loci were shared among species. For our entire dataset, we retained 1170 loci with a total 39,616 Single Nucleotide Polymorphisms (SNPs). Our sequence matrix of 91 samples had 557,972 sites with 49.80% missing sites.

**Figure 2:**
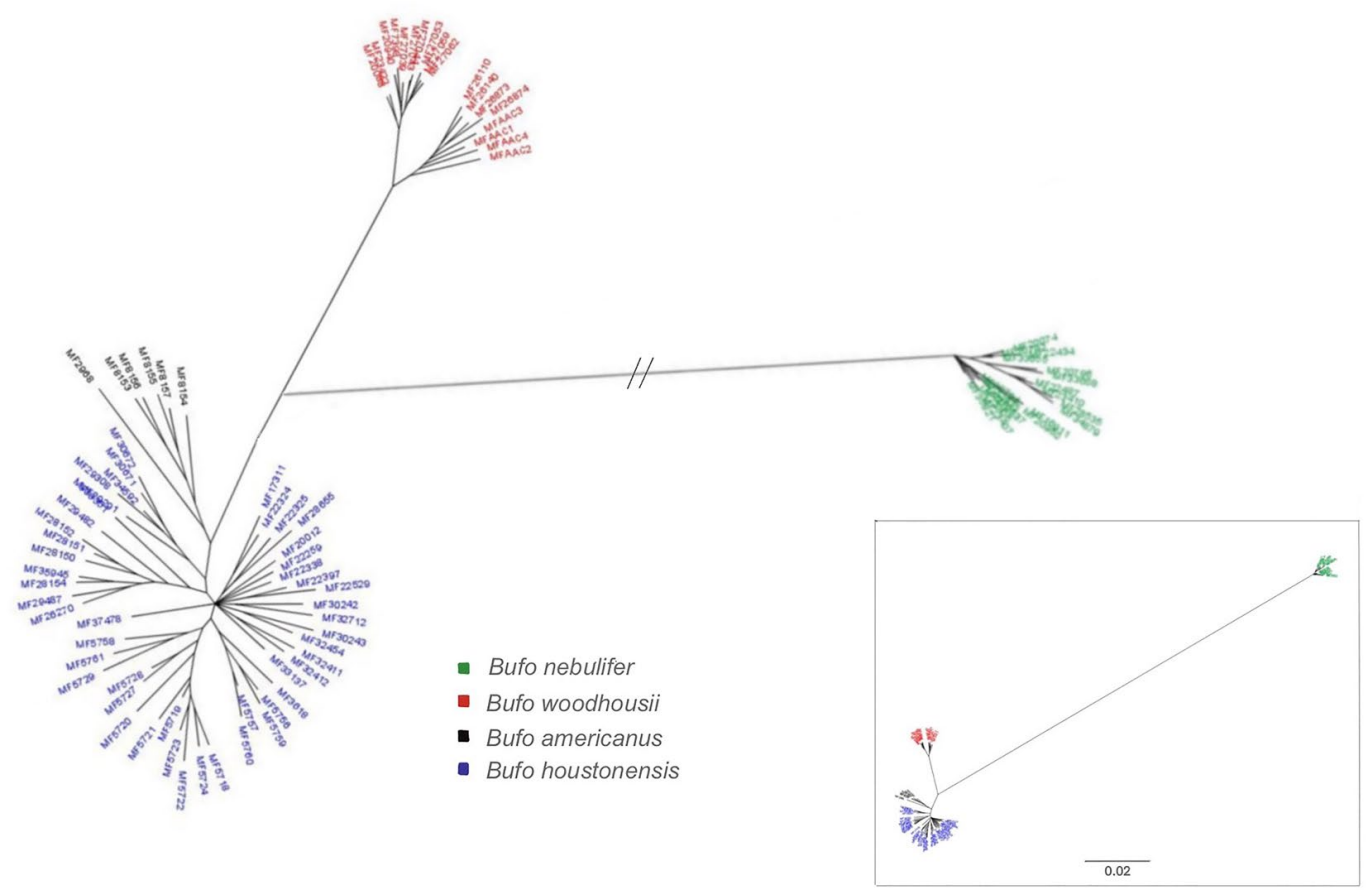
Bayesian majority rule consensus tree generated from concatenated ddRAD sequence supermatrix with 1170 loci from 91 individuals that includes 6 American Toads (*Bufo* [= *Anaxyrus*] *americanus*), 48 Houston toads (*B. houstonensis*), 18 Woodhouse’s Toads (*B. woodhousii*), and 19 Gulf Coast Toads (*B. nebulifer*). Branch lengths between Nearctic and Middle American species (denoted in green) indicate the greatest divergence. The next largest genetic divergence was between *B. woodhousii* (denoted in red) and *B houstonensis*. In comparison, *B. houstonensis* (denoted in blue) and *B. americanus* (denoted in black) were less genetically divergent but each species grouped in distinct clades*.*

The Bayesian consensus tree for our entire dataset (Fig. 2) showed the greatest genetic divergence between Nearctic and Middle American species, while the second largest genetic divergence was between *B. woodhousii* and *B. houstonensis.* In comparison, *B. houstonensis* and *B. americanus* were less genetically divergent but each species grouped in distinct clades (Fig. 2).

### Genotype structure and ancient admixture

**Figure 3:**
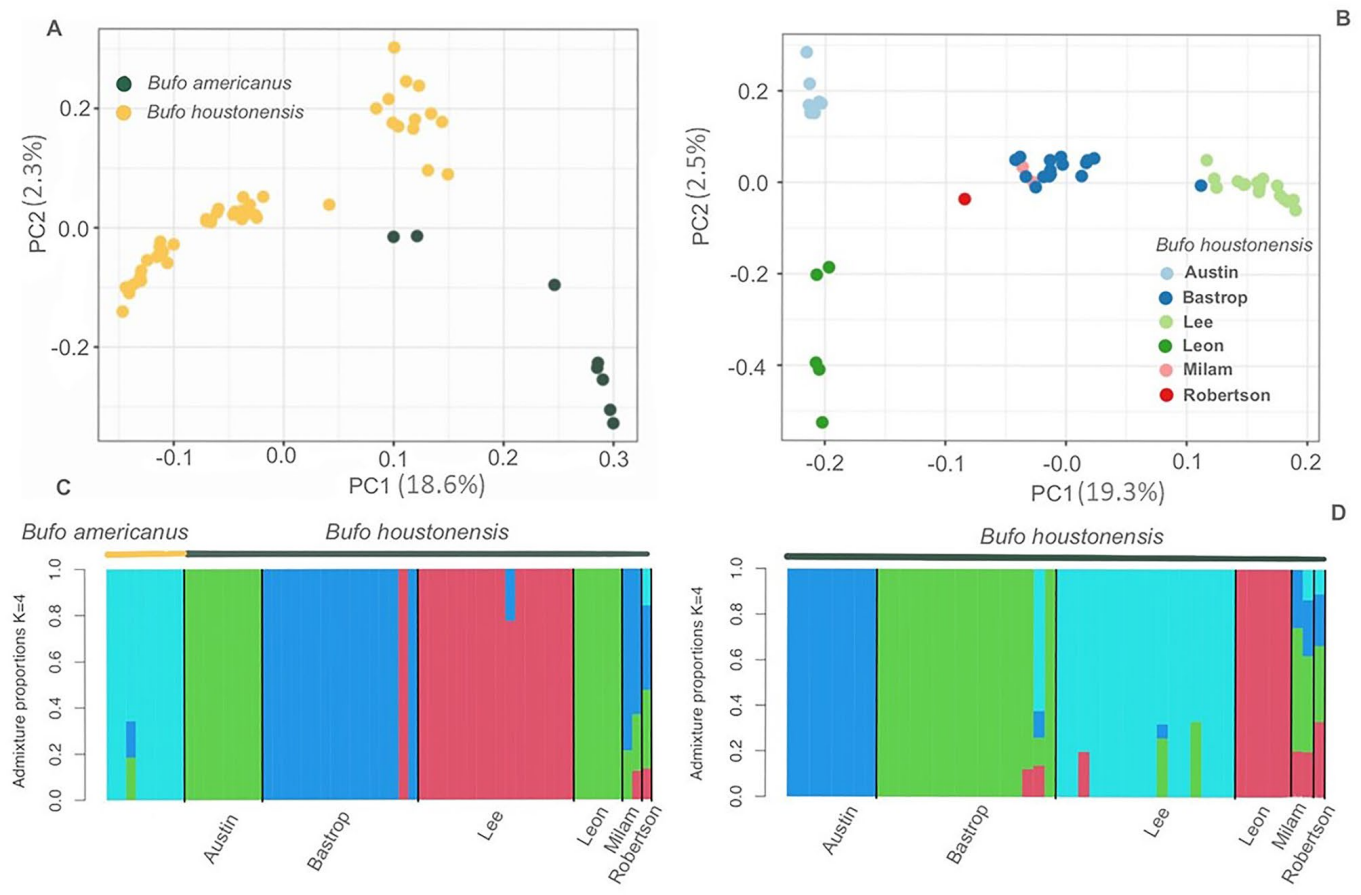
(**A**) Principal component analysis using 107,509 sites from 8 American Toads (*Bufo* [= *Anaxyrus*] *americanus*) and 48 Houston toads (*B. houstonensis*). The first two components of this PCA accounted for 20.9% of the variation in our dataset. (**B**) Principal component analysis using 92,228 sites from 48 Houston toads (*Bufo* [= *Anaxyrus*] *houstonensis*). The first two components of this PCA accounted for 21.8% of the variation in our dataset. (**C**) Admixture analysis of 8 American Toads (*Bufo* [= *Anaxyrus*] *americanus*) and 48 Houston toads (*B. houstonensis*) distinguished between *B. americanus* and *B. houstonensis*. (**D**) Admixture analysis of 48 Houston toads (*Bufo* [= *Anaxyrus*] *houstonensis*) identified 4 genomic clusters at the top level of hierarchical structure, enabling delineation of one management unit each from Bastrop, Lee, Austin, and Leon Counties.

From all individual alignments, we analyzed 151,115,496 sites and retained 292,117 sites after filtering. For the subset of *B. americanus* and *B. houstonensis* alignments, we analyzed 91,537,049 sites and retained 107,509 sites after filtering. With alignments of *B. houstonensis* alone, we analyzed 84,066,898 sites and retained 92,228 sites after filtering. We observed separation between *B. americanus* and *B. houstonensis* with the first principal component (PC1) accounting for 18.6% of total variation in our dataset while the second principal component accounted for 2.3% of total variation in our dataset. Additionally, structure within populations of *B. houstonensis* and *B. americanus* was indicated along both principal components (Fig. 3a). Similarly, we saw separation among populations of *B. houstonensis* from Bastrop, Lee, Austin, and Leon Counties, with PC1 and PC2 accounting for 19.3% and 2.5% of total variation in our dataset (Fig. 3b). Admixture analysis between *B. americanus* and *B. houstonensis* supported *K* = 4 (i.e., highest Δ*K* value; Fig. S18) grouping levels, with *B. americanus* assigning to one genomic cluster, and three other genomic clusters identified in *B. houstonensis* (Fig. 3c). Admixture analysis within *B. houstonensis* supported *K* = 4 (i.e., highest Δ*K* value; Fig. S19) grouping levels, with *B. houstonensis* from Austin, Leon, Lee, and Bastrop Counties assigning to one genomic cluster each (Fig. 3d). Individuals sampled from Milam County (*n* = 2) showed mixed ancestry from three and four genomic clusters while the lone individual sampled from Robertson County showed shared ancestry with toads from all four genomic clusters.

**Figure 4:**
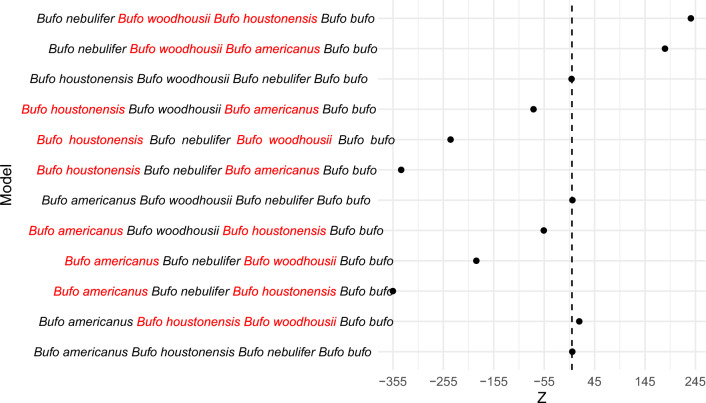
*Z*-scores from ABBA-BABA tests of ancient gene flow between populations of Nearctic American Toads (*Bufo* [= *Anaxyrus*] *americanus*), Houston toads (*B. houstonensis*), Woodhouse’s Toads (*B. woodhousii*), and Middle American Gulf Coast Toads (*B. nebulifer*). Significant gene flow between taxa is indicated in red font. We aligned sequences to a common toad (*Bufo bufo*) reference genome and assumed allele states in this reference sequence were ancestral states. We used 292,117 sites and corroborated ancient gene flow between Nearctic species (i.e., *B. americanus*, *B. houstonensis*, and *B. woodhousii*) but found no support for gene flow between Nearctic and Middle American (i.e., *B. nebulifer*) species.

In six gene flow models that used *B. americanus* as the sister group (H1), we observed a significantly higher ABBA count in one model that indicated gene flow between *B. houstonensis* (H2) and *B. woodhousii* (H3; *Z* = 14.54; Table 1, Fig. 4). We did not observe significant gene flow between *B. americanus* (H1) and *B. nebulifer* (H3) or between *B. houstonensis* (H2) and *B. nebulifer* (H3; *Z* = 0.68; Table 1, Fig. 4). Similarly, we did not find support for significant gene flow between *B. woodhousii* (H2) and *B. nebulifer* (H3; *Z* = 1.01; Table 1, Fig. 4). In the remaining three models, we observed significantly higher BABA counts. Two models indicated significant gene flow between *B. americanus* (H1) and *B. houstonensis* (H3*; Z* =  − 355.05; Table 1; Fig. 4) and *B. americanus* (H1) and *B. woodhousii* (H3; *Z* =  − 189.58; Table 1; Fig. 4) relative to *B. nebulifer* (H2). One model supported significant gene flow between *B. houstonensis* (H3; *Z* =  − 55.85; Table 1; Fig. 4) and *B. americanus* (H1) relative to *B. woodhousii* (H2).

**Table 1:** Results of ABBA-BABA tests of ancient gene flow between populations of Nearctic American Toads (*Bufo* [= *Anaxyrus*] *americanus*; AT), Houston toads (*B. houstonensis*; HT), Woodhouse’s Toads (*B. woodhousii*; WT), and Middle American Gulf Coast Toads (*B. nebulifer*; GT).

D	JK-D	V(JK-D)	Z	p value	nABBA	nBABA	nBlocks	H1	H2	H3	H4
0.004	0.004	4.1E−05	0.68	0.49	15,055	14,924	1060	AT	HT	GT	CT
0.08	0.08	2.9E−05	14.5	< 0.001	56,864	48,555	1150	AT	**HT**	**WT**	CT
− 0.83	− 0.83	6E−06	− 355.1	< 0.001	15,055	166,312	1075	**AT**	GT	**HT**	CT
− 0.75	− 0.75	1.5E−05	− 189.6	< 0.001	13,037	89,282	1054	**AT**	GT	**WT**	CT
− 0.31	− 0.31	3.1E−05	− 55.9	< 0.001	56,864	108,079	1155	**AT**	WT	**HT**	CT
0.008	0.008	5.5E−05	1.01	0.31	13,037	12,843	1042	AT	WT	GT	CT
− 0.84	− 0.84	6.0E−06	− 338.6	< 0.001	14,924	166,312	1076	**HT**	GT	**AT**	CT
− 0.74	− 0.74	9.0E−06	− 240.6	< 0.001	25,649	169,765	1091	**HT**	GT	**WT**	CT
− 0.38	− 0.38	2.5E−05	− 76.2	< 0.001	48,555	108,079	1155	**HT**	WT	**AT**	CT
− 0.004	− 0.004	2.5E−05	− 0.75	0.45	25,649	25,844	1071	HT	WT	GT	CT
0.75	0.75	1.6E−05	184.5	< 0.001	89,282	12,843	1056	GT	**WT**	**AT**	CT
0.73	0.73	1.0E−05	235.8	< 0.001	169,765	25,844	1091	GT	**WT**	**HT**	CT

We aligned sequences to a common toad (*Bufo bufo*) reference genome and assumed allele states in this reference sequence were ancestral states. We used 292,117 sites and corroborated ancient gene flow between Nearctic species (i.e., *B. americanus*, *B. houstonensis*, and *B. woodhousii*) but no support for gene flow between Nearctic and Middle American (i.e., *B. nebulifer*) species. D: D statistic [i.e., (nABBA-nBABA)/(nABBA + nBABA)]; JK-D: Jackknife estimate of D statistic, V (JK-D): Standard deviation of Jackknife estimate; Z: *Z*-score.

Significant values are in bold.

Three of the four models that used *B. houstonensis* as the sister group (H1) showed significantly higher BABA counts. We found support for significant gene flow between *B. americanus* (H3; *Z* =  − 338.61; Table 1, Fig. 4) and *B. houstonensis* (H1) as well as between *B. woodhousii* (H3; *Z* =  − 240.64; Table 1, Fig. 4) and *B. houstonensis* (H1) relative to *B. nebulifer* (H2). Significant gene flow was also supported between *B. houstonensis* (H1) and *B. americanus* (H3; *Z* =  − 76.21; Table 1; Fig. 4) relative to *B. woodhousii* (H2). We did not find support for significant gene flow between *B. houstonensis* (H1) and *B. nebulifer* (H3; *Z* =  − 0.76; Table 1; Fig. 4) or between *B. woodhousii* (H2) and *B. nebulifer* (H3; Table 1; Fig. 4).

We saw significantly higher ABBA counts in models with *B. nebulifer* as the sister group (H1), wherein significant gene flow was indicated between *B. houstonensis* (H3; *Z* = 235.78; Table 1; Fig. 4) and *B. woodhousii* as well as between *B. americanus* (H3; *Z* = 184.47; Table 1; Fig. 4) and *B. woodhousii*.

## Discussion

We observed uneven sequencing coverage with heterogeneity in input among samples and variation in locus coverage. Multiple sources such as variable recovery during library preparation, insufficient sequencing coverage, and mutations at enzyme recognition sites have been attributed to missing RADseq data^26–29^. Data sets generated using restriction enzymes with recognition sites that are 6-bp long result in a larger number of fragments relative to when using restriction enzymes with recognition sites that are 8-bp in length^27^. Empirical data suggest that the number of loci shared among taxa in 6-bp cutter data sets are better predicted by sequencing coverage than phylogenetic distance, suggesting such data sets may be consistently under-sequenced^27^. We acknowledge uneven and low depth of sequencing is a source of missing data for our study. However, we also observed hierarchical data loss in our study, with a strong inverse correlation between number of shared loci and phylogenetic distance. Since our missing data were phylogenetically distributed^27^, we used computationally tractable, sparse supermatrices of concatenated ddRAD data to potentially increase resolution in our phylogenetic trees^30,31^ relative to prior taxonomic assessments.

Previous mtDNA based studies that included constituents of the *Bufo americanus* species group have inferred non-traditional relationships among *B. americanus* and *B. houstonensis*. Pauly et al.^24^ inferred *B. woodhousii* was sister to a clade that included *B. americanus*, *B. houstonensis*, and *B. velatus,* with monophyly of mitochondrial haplotypes in *B. a. charlesmithii*, *B. houstonensis*, and *B. velatus* to the exclusion of *B. a. americanus*. Goebel et al.^23^ inferred similar results with *B. woodhousii* grouping sister to *B. hemiophrys* which in turn was sister to a clade that included *B. houstonensis* and *B. americanus*. The Bayesian Consensus tree for our entire dataset assigned *B. houstonensis* and *B. americanus* to separate groups. Further, lower genetic divergence was observed between *B. americanus* and *B. houstonensis* relative to the divergence of each from a clade of *B. woodhousii*. These results support the identity of *B. houstonensis* as a taxon that is distinct from, but likely derived from a precursor of, present-day *B. americanus*. We observed geographically concordant structure within *B. houstonensis*. Toads from Bastrop and Lee Counties grouped sister to each other, albeit with low (< 70%) support and with five of 16 samples from Bastrop County appearing unresolved within this grouping. Samples from *B. houstonensis* populations in Austin and Leon grouped distinct from each other and from toads sampled in Bastrop and Lee Counties. Within a clade of *B. americanus*, the single Eastern American toad (*B. americanus americanus*) from New York grouped sister to Dwarf American toads (*B. americanus charlesmithii*) from Missouri.

Additionally, our results also supported genetic structure within the clade of *B. woodhousii*. Toads from the South-Central Plains in Oklahoma (i.e., Southeast Oklahoma) and Post Oak Savannah region in Texas (i.e., Northeast Texas) formed one group, while *B. woodhousii* from Blackland Prairie (i.e., North and Central Texas), High Plains (i.e., Northwest Texas), and Central Great Plains in Oklahoma (i.e., North and Central Oklahoma) formed a second group. We lack the data to provide insight into reasons for the observed structure within *B. woodhousii*. Given that *B. woodhousii* has a broad distribution^32^, we suggest prioritizing efforts to characterize genetic variation in the species to identify management units of evolutionary and potential conservation significance. Finally, the greatest genetic divergence in our dataset was between Nearctic taxa and *B. nebulifer*. However, we observed low support (< 70%) for within group structure in *B. nebulifer*, which can be attributed to a low number of loci available within constituents of this clade.

Supermatrices of concatenated ddRAD sequence data enable effective tests of evolutionary hypotheses^27,31^. However, we observed low and uneven sequencing coverage across samples in our dataset. Given such low coverage in our dataset, we sought to also use an approach that avoided basing downstream analysis on raw counts of sequenced bases or called genotypes^33^. For this reason, we also incorporated a probabilistic approach that used genotype likelihoods to ascertain genetic distinctiveness between *B. houstonensis* and *B. americanus*, determine genetic structure within *B. houstonensis*, and test for historic gene flow among constituent taxa in our study. We observed two *B. americanus* individuals were clustered in the center of the coordinate space of our PCA plot- indicating these were either different from the remainder of *B. americanus* (*n* = 6) in our study or more likely, that these samples clustered in the middle due to a low number of input reads and sequencing coverage. However, admixture analyses clearly corroborate the separation of *B. americanus* and *B. houstonensis* into distinct clusters while also indicating population genetic structure within *B. houstonensis*. Reproductive isolation of *B. houstonensis* from *B. americanus* corroborates prior phylogenetic support for the species identity of *B. houstonensis*.

Using genotypes from ten polymorphic microsatellite markers in 439 individual *B. houstonensis* from six counties in Texas, McHenry observed two clusters (i.e., North and South) at the uppermost level of hierarchical structure. Both clusters occurred in all counties, except Austin County in which only one cluster (i.e., North) was seen^16^. Second order analyses indicated at least nine clusters at varying levels of genetic differentiation. Five of these unique clusters were detected in Bastrop County alone and the remaining four were one cluster each from Austin, Milam, Colorado, and Leon Counties^16^. Sampling bias was a likely influence in McHenry’s study as 95% of all *B. houstonensis* samples were detected in Bastrop County, where the largest known population occurs^16,19^. We used genome-wide DNA sequence data with a smaller proportion (33%) of *B. houstonensis* from Bastrop County. Since we sought comparison with McHenry’s^16^ characterization of population genetic structure, 40% of our *B. houstonensis* samples were also used in her study. At the uppermost level of hierarchical structure for *B. houstonensis* populations in our study, we observed four unique genomic clusters. Toads from Austin, Leon, Lee, and Bastrop Counties were distinct from each other. Additionally, individual toads from Milam County showed mixed ancestry from three and four genomic clusters while the single available sample from Robertson County showed shared ancestry from all four genomic clusters. This is a sampling artefact, since these localities were represented by only one or two individuals.

Despite low sequencing coverage, the genome-wide DNA sequence data used in this study provides considerable power in detecting population genetic structure within *B. houstonensis*. We recognize four Management Units (MUs) in *B. houstonensis* and thus, conservation actions for species recovery need to be specific for each MU. Population supplementation to raise juvenile survivorship and habitat restoration to enhance viability of additional subpopulations have been identified as primary measures to reduce extinction risk for *B. houstonensis*^34^. Further, an inherently low juvenile survival in *B. houstonensis*^35,36^ requires scaled-up investment in captive propagation efforts for population supplementation to be successful^34^. These conservation actions while expensive are warranted, given the endangered status of this relict species^7,8,34^. Further, given populations in each county (i.e., Austin, Leon, Lee, and Bastrop) are distinct from each other, we suggest that captive cohorts meant for supplementation need to be of the same provenance as resident populations. The current genetic clustering results are indicative of low levels of gene flow between populations*,* which contrasts with McHenry’s^16^ suggestions that *B. houstonensis* was historically larger and more contiguously distributed with few populations in complete isolation.

McHenry’s^16^ hypothesis was based on an overall high genetic diversity as evidenced by a considerable number of mitochondrial haplotypes ($n=14$) and microsatellite alleles per locus (*n* = 8–29) across the range of *B. houstonensis*^16^. Further, some of these mitochondrial haplotypes and microsatellite alleles were detected range-wide^16^. Models based on coalescent theory are required to characterize population-level processes (e.g., demographic changes, migration, and isolation) that have resulted in the genetic structure we observed within *B. houstonensis*^37^. However, within the context of historic processes, we tested our dataset for signs of historical admixture events. Gene flow between pairs of Nearctic study species was significant. Using *Z* scores as indices of strength of gene flow, we observed the strongest support for gene flow between *B. houstonensis* and *B. americanus*. Second, gene flow between *B. houstonensis* and *B. woodhousii* received stronger support relative to gene flow between *B. americanus* and *B. woodhousii*. Natural hybridization has been previously documented among members of the *Bufo americanus* species group and potentially introduced novel genetic variation at the beginning of a radiation of these toads^16,20,21,38^. Fontenot et al.^20^ observed high levels of gene flow among pairs in the *Bufo americanus* species group that occurred in geographic proximity and shared similar male advertisement calls. The substantial signal of ancient gene flow between *B. americanus* and *B. houstonensis* may be concordant with a pattern of erstwhile range expansions and secondary contact between species pairs that were geographically proximate and share similar advertisement calls prior to complete reproductive isolation^17,20^.

Gene flow models did not indicate historic admixture events between *B. nebulifer* and any Nearctic study species. However, hybridization events between *B. houstonensis* and *B. nebulifer* as well as between *B. woodhousii* and *B. nebulifer* have been documented^16,39^. McHenry conducted a range-wide genetic assessment of *B. houstonensis* for interspecific admixture with *B. nebulifer* and estimated that nearly 10% of all sampled *B. houstonensis* were admixed^16^. Thus, hybridization between *B. houstonensis* and *B. nebulifer* can be characterized as an added threat to persistence of the former species. Gene flow models in this study do not support any historic context for hybridization between Nearctic toad taxa and *B. nebulifer*, possibly due to reproductive isolating mechanisms such as offset breeding times and differences in preferred habitat^16,22^. Hybridization of Nearctic taxa with *B. nebulifer* may be more contemporaneous and due to the disruption of ecological barriers and temporal isolation in breeding activity consequent to human-induced land-use and climate change^16,22,40^. We draw attention to the potential of such hybridization as a contemporaneous driver of extinction in endangered Nearctic toads (e.g., *B. houstonensis*). We suggest population-level genetic assessments for Nearctic toads that are sympatric with *B. nebulifer* to determine whether interspecific admixture poses a threat to species persistence.

Historically, the taxonomic status of *B. houstonensis* has remained uncertain^9,16,19^. Our study clarified that *B. houstonensis* is a species distinct from *B. americanus* via a phylogenetic analysis of a concatenated sequence matrix and an admixture analysis that used genotype likelihoods. An admixture analysis that used genotype likelihoods identified a minimum of four genetically distinct areas or populations (i.e., MUs) within *B. houstonensis*. These included eastern populations of *B. houstonensis* from Austin and Leon, as well as western populations from Bastrop and Lee Counties. Given that *B. houstonensis* is a distinct species with a high risk of extinction, conservation measures such as habitat restoration to increase viability of additional subpopulations and population supplementation to raise juvenile survival are warranted^34–36^. We suggest that captive propagation and population supplementation to raise juvenile survival need to be cognizant of the underlying genetic structure observed within *B. houstonensis*. Our study strengthens the need and ability for *B. houstonensis* conservation while re-affirming the evolutionary novelty of an endangered relict.

## Methods

### Sampling and DNA extraction

We included 48 *B. houstonensis* sampled from 2001 to 2015 throughout much of their geographic range (Fig. 1, Table S1). We specifically chose *B. houstonensis* samples from or prior to 2015 since these preceded full-scale population supplementation efforts^41^. We had a lower number of samples available for *B. americanus*. We used 8 samples of *B. americanus* collected from 1996 to 2003, that included 6 samples of Dwarf American toads (*B. americanus charlesmithii*) and 2 samples of Eastern American toads (*B. americanus*; Table S1). We included 18 *B. woodhousii* sampled across much of their range in Texas, USA from 2002 to 2015 and 19 *B. nebulifer* sampled in Texas, USA and Tamaulipas, Mexico from 2002 to 2014 (Table S1). We specifically included *B. nebulifer* due to previously documented hybridization with *B. houstonensis*^16,39^. All tissue samples were stored in 95% ethanol and afterwards at − 80 °C for long- term storage. Tissue samples were deposited in the Michael R. J. Forstner Frozen Tissue catalog currently held at Texas State University, San Marcos, Texas.

For each putative species we sought to sample the largest spatial extent of species’ range, within the constraints of sample availability. Since we had samples from throughout most of the range of *B. houstonensis* relative to other study species, the number of samples for the former is considerably larger. However, we sought to exceed a minimum of four samples per taxon. We used a lower number of samples relative to prior studies^16^ but attempted to offset this with a higher number of markers per sample.

Sample localities for *B. woodhousii* were both sympatric and allopatric to *B. houstonensis* and *B. americanus*, while localities for *B. nebulifer* were both sympatric and allopatric to *B. houstonensis* and *B. woodhousii* (Fig. 1). Sample collection from toads was authorized under Federal Fish and Wildlife Permits (TE039544-0, TE039544-1, TE039544-2, TE004472-0, and TE004472-1), Texas Parks and Wildlife Department Scientific Research Permits (SPR-0102-191, SPR-0290-022). Our experimental protocol was approved by the Institutional Animal Care and Use Committee at Texas State University via permits 5Qrs45_02, HGVMAD_02, 04-0485904A30, 0713_0428_07, 0810_0208_11, and IACUC 201648186. Collection and transport of *B. nebulifer* samples from Mexico were authorized under CITES Permit Number 05US704066/9 and Costa Rica MINAE Resolucion Numbers 237-98-OFAU and 019-2000-OFAU (Collecting Licenses 0023073 and 0205-00). All procedures were performed in accordance with the relevant guidelines and regulations of each institution and where applicable, in accordance with ARRIVE guidelines (https://arriveguidelines.org/).

Tissue samples included toe-clips or blood tissue from adult toads and tail-clips from tadpoles. All tadpoles used in this study were the only available samples from Lee County. Individual tadpoles were sampled from three different ponds and likely belonged to different cohorts as evidenced by Gosner stage of development. DNA was extracted from samples in ethanol and lysis buffer using Qiagen DNeasy Blood and Tissue kits following manufacturer protocol. High-molecular weight DNA extracted from samples was examined for quality on agarose gels and quantified with a Qubit version 2.0 fluorometer (Thermo Fisher Scientific).

### ddRADseq data collection

Genomic libraries were prepared and sequenced at the University of Texas Genomic Sequencing and Analysis Facility (Austin, TX). The ddRADseq data were collected using the protocol described by Peterson et al.^42^. Samples were normalized to 10 ng/uL gDNA and ~ 100 ng of gDNA were digested with 1 unit each of EcoRI and MspI (at 10,000×) in a single reaction with the manufacturer recommended buffer (New England Biolabs) for 2 h at 37 °C. Samples were purified with AMPure XP beads (Beckman Coulter Life Sciences) prior to ligation of barcoded Illumina adapters onto the fragments. Oligonucleotide sequences used in barcoding and adding Illumina indices to fragments were per Peterson et al.^42^. Prior to pooling, samples were purified with AMPure XP beads to enrich for a broad size range (150–800 bp). Equimolar amounts of individual libraries were pooled to constitute four libraries. Pooled libraries were size selected over a narrow range (540–660 bp after accounting for adapter length) using a Pippin Prep (Sage Science) size fractionator. PCR amplification of size selected DNA was performed using 1 μL each of 25 μM Illumina dual index primers, 23 μL of size selected DNA, and 25 μL of NEBNext High-Fidelity 2× PCR master mix (New England Biolabs). PCR was performed with an initial denaturation at 98 °C for 30 s, followed by 12 cycles each consisting of denaturing at 98 °C for 10 s, annealing at 65 °C for 30 s, and extension at 72 °C for 30 s, and a final extension step at 72 °C for 5 min. Subsequently, samples were purified using AMPure XP beads (Beckman Coulter Life Sciences) and each library was sequenced, in two replicate runs, on a single lane of an Illumina Miseq platform under a 300 bp paired-end read protocol (2 $\times$ 300 bp).

### De novo assembly and phylogenetic analyses

We processed raw Illumina reads with ipyrad version 0.9.59^43^. We demultiplexed samples using unique inline barcodes, allowing no mismatches, for each sample and trimmed reads for adapter contamination. Sites with accuracy of base calls under 99% (Phred Quality Score < 20) were assigned ‘N’ characters and reads with ≥ 5 N’s were discarded. Within the ipyrad pipeline, read pairs were merged and clustered with VSEARCH^44^ and subsequently aligned with MUSCLE^45^.

Clustering thresholds within the ipyrad pipeline are used to establish homology among reads within samples and identify putative orthologs among samples^46^. We diagnosed the optimal clustering threshold value by examining the fraction of inferred paralogous clusters, individual heterozygosity, total number of SNPs, and correlation between individual pairwise data-missingness and genetic distance across ten separate runs with clustering thresholds that ranged from 0.50 to 0.99 usually in steps of 0.05 (e.g., 0.50 to 0.55)^47^. We repeated an examination of clustering thresholds that ranged from 0.90 to 0.99 but in steps of 0.01 while using fraction of inferred paralogs, individual heterozygosity, and total number of SNPs as diagnostic parameters. For runs that increased in steps of ~ 0.05, we used a minimum sequencing depth of 2, based on a relatively low number of raw reads per sample, and a minimum of 7 samples (~ 8%) having data to retain a locus. For ipyrad runs that increased in steps of 0.01, we used a minimum sequencing depth of 2 and a minimum of 15 samples (~ 16%) having data to retain a locus. We used default values to control alignment quality by excluding consensus sequences with ≥ 5% ambiguous and heterozygous bases and excluding loci with ≥ 20% SNPs and ≥ 8 indels. Additionally, we used default values to detect and filter paralogous clusters by allowing a heterozygous site to occur in a maximum of 50% of samples. Among clustering thresholds that increased in broad steps (i.e., ~ 0.05), we determined a clustering threshold of 0.95 as optimal since this minimized the fraction of inferred paralogous clusters, maximized individual heterozygosity and total number of SNPs, and was the threshold value (Figs. S1–S12) at which the correlation between pairwise data-missingness and genetic distance steeply increased^47^. Similarly, while examining clustering thresholds at narrow consecutive increases (i.e., steps of 0.01) we determined 0.95 as the optimal clustering threshold as this minimized the fraction of inferred paralogous clusters, maximized individual heterozygosity, and the total number of SNPs (Figs. S13–S15).

We generated an output file in custom format (i.e., *.loci) in ipyrad and used Matrix Condenser to visualize the effects of excluding samples with poor coverage and changing the minimum number of samples to retain a locus^43,48,49^. We excluded two samples with fewer than 10,000 reads and greater than 95% missing data. One of these samples (MF1103, Table S1) was an eastern American toad (*B. americanus americanus*) from New York State and the other (MF7399, Table S1) was a dwarf American toad (*B. americanus charlesmithii*) from Oklahoma. We also retained loci that were recovered for a minimum of 41 samples, with 49.8% missing sites in our sequence matrix for 91 samples. Despite a more stringent filtering threshold, we continued to see a systematic pattern to missing data—with few loci overlapping between Middle American and Nearctic species (Fig. S16).

We used AIC and BIC scores in ModelTest-NG^50^ to select the best-fit DNA substitution model for concatenated ddRAD loci in our final dataset. We evaluated 24 candidate models across three DNA substitution schemes (i.e., JC, HKY, and GTR) and four rate-heterogeneity models (i.e., Uniform, Invariant, Gamma, Gamma + Invariant). Bayesian analyses were conducted in MrBayes 3.2.7^51^ to generate phylogenetic hypotheses from matrices of concatenated loci for 91 samples. We conducted two independent runs of 3 × 10^6^ generations from random starting trees using four Markov chains (one cold, three heated, temperature of 0.01) sampling every 1000 generations. We used the average standard deviation of split frequencies (ASDF) and potential scale reduction factor (PSRF) as convergence diagnostics, with analyses run until ASDF $=$ 0.10 and PSRF approached 1.0. We discarded 25% of the samples as burn-in and used the remaining samples to compute a majority consensus tree. We used a *B. nebulifer* sequence as an outgroup to Nearctic taxa included in our study. We visualized the Bayesian consensus tree from this phylogenetic analysis in FIGTREE, version 1.4.4 (http://tree.bio.ed.ac.uk/software/figtree).

### Probabilistic genomic analysis, genotype structure, and ancient admixture

We aligned 48 *B. houstonensis* and 8 *B. americanus* sequences processed in ipyrad^43^ to a common toad (*Bufo bufo*) reference genome (5.04 Gb in 1307 sequence scaffolds, GenBank assembly accession no.GCA_905171765.1) using the local algorithm set to default parameters in Bowtie2^52^. A majority (99.1%) of this congeneric reference genome was assigned to 11 chromosomal-level scaffolds and represents the closest related high quality genomic resource that was available^53^. We discarded reads with more than one match to the genome and converted alignments to indexed BAM format using SAMtools^54^. We merged alignments from each sequencing run and subsequently removed duplicates using Picard Tools. We used the probabilistic framework implemented in Analysis of Next Generation Sequencing Data (ANGSD) for population genetic analysis. Given low sequencing coverage in our dataset, we sought to also use an approach that avoided basing downstream analysis on raw counts of sequenced bases or called genotypes^33^. We generated beagle likelihood files (doGlf 2) in ANGSD while using a minimum mapping quality score (minMapQ) of 20, minimum base quality score (minQ) of 25, SAMtools genotype likelihood model (GL1), and fixed major and minor allele frequencies (doMaf 1). Further, we designated polymorphic sites at Minor Allele Frequency (MAF) > 0.05 and probability of site not being polymorphic < 1 × 10^–6^. Downstream analyses (e.g., NgsAdmix) estimated composite likelihoods that were robust to non-independence of sites^55^.

We assessed relatedness among tadpoles collected from Lee County, prior to including these samples in downstream analyses. We used IBSrelate to identify pairs of related individuals without requiring population allele frequencies and generated KING-robust kinship coefficient estimates for every pair^56^. We observed negative estimates of kinship coefficients across pairs (Fig. S17) and concluded there was no relatedness among pairs, enabling inclusion of these samples for population genetic analyses. We examined the overall quality of discovered SNPs and their reliability in distinguishing between *B. houstonensis* and *B. americanus* as well as among populations (sensu McHenry, 2010) of *B. houstonensis* by conducting a Principal Component Analysis (PCA) of genetic structure in the ngsCovar module of ngsTools^57^. We also investigated genetic structure between *B. houstonensis* and *B. americanus* as well as among populations of *B. houstonensis* (sensu McHenry, 2010) by calculating individual admixture proportions at different grouping levels (1–10) using NgsAdmix^55^. For each between and within-species admixture analyses, we used 10 independent runs of 20,000 iterations and determined the most likely grouping level (*K*) per Evanno et al.^58^.

We also sought to detect historical gene flow between *B. houstonensis, B. americanus, B. woodhousii,* and *B. nebulifer* and performed the ABBA-BABA test of ancient admixture (or wrong tree topology) in ANGSD^59^. This provides a way to test the correctness of a hypothetical genetic relationship among four groups. We examined distribution of ancestral (‘A’) and derived (‘B’) alleles across the genomes of four groups of individuals that include an ancestral outgroup. Two allelic patterns ‘ABBA’ or ‘BABA’ should occur equally frequently under a scenario with no introgression. An excess of either ‘ABBA’ or ‘BABA’ would indicate gene flow between two taxa^59^. We aligned all individual sequences in each group to the *Bufo bufo* reference genome and determined polymorphic sites in ANGSD, as described previously. We assessed the distribution of ancestral (‘A’) and derived (‘B’) alleles within taxa wherein allele states in *Bufo bufo* were considered ancestral states and all bases at each position were considered (doAbbababa2 1). We evaluated 12 gene flow models for ancient admixture between sister Nearctic representatives as well as between Middle American and Nearctic species. We set *B. americanus, B. houstonensis*, and *B. nebulifer* each as the sister group (H1) in 6, 4, and 2 models, respectively. The number of ABBA and BABA counts and Patterson’s D-statistic were calculated without error correction and ancient transition removal. Significance of the D-statistic was assessed with *Z* values from a block Jackknife procedure implemented in the module^59^.

## Supplementary Information

Supplementary Information.

## Data Availability

Nexus files used in phylogenetic analyses, XML files used in BEAST analyses, and individual alignment files (bam files) used in probabilistic genomic analyses are available in the Dryad Digital Repository (10.5061/dryad.m37pvmd52). All scripts used in phylogenetic and population genetic analysis will be made available by Shashwat Sirsi (s_s477@txstate.edu) on request.

## References

[1] Howard, S. D. & Bickford, D. P. Amphibians over the edge: Silent extinction risk of data deficient species. *Divers. Distrib.* **20**, 837–846 (2014).

[2] Stuart, S. N. *et al.* Status and trends of amphibian declines and extinctions worldwide. *Science* **306**, 1783–1786 (2004). 15486254 10.1126/science.1103538

[3] Kiesecker, J. M., Blaustein, A. R. & Belden, L. K. Complex causes of amphibian population declines. *Nature* **410**, 681–684 (2001). 11287952 10.1038/35070552

[4] Lips, K. R., Burrowes, P. A., Mendelson, J. R. III. & Parra-Olea, G. Amphibian declines in Latin America: Widespread population declines, extinctions, and impacts. *Biotropica* **37**, 163–165 (2005).

[5] Sodhi, N. S. *et al.* Measuring the meltdown: Drivers of global amphibian extinction and decline. *PLoS ONE* **3**, e1636 (2008). 18286193 10.1371/journal.pone.0001636 PMC2238793

[6] Brown, L. E. The status of the near-extinct Houston toad (*Bufo houstonensis*) with recommendations for its conservation. *Herpetol. Rev.* **6**, 37–40 (1975).

[7] Gottschalk, J. S. United States list of endangered native fish and wildlife. *Fed. Regist.* **35**, 16047–16048 (1970).

[8] Honegger, R. E. *Red Data Book Vol. 3 Amphibia and Reptilia.* (International Union for Conservation of Nature and Natural Resources, 1970).

[9] Blair, W. F. *Bufo* of North and Central America. In *Evolution in the Genus Bufo* (ed. Blair, W. F.) 93–101 (University of Texas Press, 1972).

[10] Blair, W. F. *Evolution in the Genus Bufo* (University of Texas Press, 1972).

[11] United States Fish and Wildlife Service. *Houston Toad (Bufo houstonensis) 5-Year Review: Summary and Evaluation*. https://www.fws.gov/southwest/es/documents/r2es/houstontoad_5-yr_review_nov2011.pdf (Accessed 12 December 2023) (United States Fish and Wildlife Service, 2011).

[12] Brown, L. E. & Mesrobian, A. Houston toads and Texas politics. In *Amphibian Declines* (ed. Lannoo, M. J.) 150–167 (University of California Press, 2005).

[13] Buzo, D. *A GIS Model for Identifying Potential Breeding Habitat for the Houston Toad (Bufo houstonensis*). M.S. Thesis, Texas State University, San Marcos (2008).

[14] Kennedy, J. Spawning season and experimental hybridization of the Houston toad, *Bufo houstonensis*. *Herpetologica* **17**, 239–245 (1962).

[15] Sanders, O. A new species of toad, with a discussion of morphology of the bufonid skull. *Herpetologica* **9**, 25–47 (1953).

[16] McHenry, D. *Genetic Variation and Population Structure in the Endangered Houston Toad in Contrast to Its Common Sympatric Relative, the Coastal Plain Toad*. Doctoral Dissertation, University of Missouri, Columbia (2010).

[17] Blair, W. F. Evolutionary relationships of North American toads of the genus *Bufo*: A progress report. *Evolution* **17**, 1–16 (1963).

[18] Brown, L. E. *Bufo houstonensis*. In *Catalogue of American Amphibians and Reptiles* (ed. Zweifel, R. G.) 131–133 (University of Texas Press, 1973).

[19] Potter, F. E., Brown, L. E., McClure, W. L., Scott, N. J. & Thomas, R. A. *Recovery Plan for the Houston Toad (Bufo houstonensis)* (United States Fish and Wildlife Service, 1984).

[20] Fontenot, B. E., Makowsky, R. & Chippindale, P. T. Nuclear-Mitochondrial discordance and gene flow in a recent radiation of toads. *Mol. Phylogenet. Evol.* **59**, 66–80 (2011). 21255664 10.1016/j.ympev.2010.12.018

[21] Masta, S. E., Sullivan, B. K., Lamb, T. & Routman, E. J. Molecular systematics, hybridization, and phylogeography of the *Bufo americanus* complex in Eastern North America. *Mol. Phylogenet. Evol.* **24**, 302–314 (2002). 12144763 10.1016/s1055-7903(02)00216-6

[22] Vogel, L. S. & Johnson, S. G. Estimation of hybridization and introgression frequency in toads (genus: *Bufo*) using DNA sequence variation at mitochondrial and nuclear loci. *J. Herpetol.* **42**, 61–75 (2008).

[23] Goebel, A. M., Ranker, T. A., Corn, P. S. & Olmstead, R. G. Mitochondrial DNA evolution in the *Anaxyrus boreas* species group. *Mol. Phylogenet. Evol.* **50**, 209–225 (2009). 18662792 10.1016/j.ympev.2008.06.019

[24] Pauly, G., Hillis, D. M. & Cannatella, D. C. The history of a Nearctic colonization: Molecular phylogenetics and biogeography of the Nearctic toads (*Bufo*). *Evolution* **58**, 2517–2535 (2004). 15612295 10.1111/j.0014-3820.2004.tb00881.x

[25] Després, L. One, two or more species? Mitonuclear discordance and species delimitation. *Mol. Ecol.* **28**, 3845–3847 (2019). 31515862 10.1111/mec.15211

[26] Cariou, M., Duret, L. & Charlat, S. Is RAD-seq suitable for phylogenetic inference? An in-silico assessment and optimization. *Ecol. Evol.* **3**, 846–852 (2013). 23610629 10.1002/ece3.512 PMC3631399

[27] Eaton, D. A., Spriggs, E. L., Park, B. & Donoghue, M. J. Misconceptions on missing data in RAD-seq phylogenetics with a deep-scale example from flowering plants. *Syst. Biol.* **66**, 399–412 (2017). 27798402 10.1093/sysbio/syw092

[28] Escudero, M., Eaton, D. A., Hahn, M. & Hipp, A. L. Genotyping-by-sequencing as a tool to infer phylogeny and ancestral hybridization: A case study in *Carex* (Cyperaceae). *Mol. Phylogenet. Evol.* **79**, 359–367 (2014). 25010772 10.1016/j.ympev.2014.06.026

[29] Huang, H. & Knowles, L. L. Unforeseen consequences of excluding missing data from next-generation sequences: Simulation study of RAD sequences. *Syst. Biol.* **65**, 357–365 (2016). 24996413 10.1093/sysbio/syu046

[30] Leache, A. D. *et al.* Phylogenomics of phrynosomatid lizards: Conflicting signals from sequence capture versus restriction site associated DNA sequencing. *Genome Biol. Evol.* **7**, 706–719 (2015). 25663487 10.1093/gbe/evv026 PMC5322549

[31] Wagner, C. E. *et al.* Genome-wide RAD sequence data provide unprecedented resolution of species boundaries and relationships in the Lake Victoria cichlid adaptive radiation. *Mol. Ecol.* **22**, 787–798 (2013). 23057853 10.1111/mec.12023

[32] Stebbins, R. C. *A Field Guide to Western Reptiles and Amphibians* 3rd edn. (Houghton Mifflin, 2003).

[33] Korneliussen, T. S., Albrechtsen, A. & Nielsen, R. ANGSD: Analysis of next generation sequencing data. *BMC Bioinform.* **15**, 356 (2014). 10.1186/s12859-014-0356-4 PMC4248462 25420514

[34] Forstner, M. R. J. & Crump, P. Houston toad population supplementation in Texas, USA in global re-introduction perspectives: 2011. In *More Case Studies from Around the Globe* (ed. Soorae, P. S.) 71–76 (IUCN/SSC Re-introduction Specialist Group & Environment Agency, 2011).

[35] Greuter, K. *Survivorship and Growth in the Endangered Houston Toad*. Master’s Thesis. Texas State University, San Marcos (2004)

[36] Swannack, T. M., Grant, W. E. & Forstner, M. R. J. Projecting population trends of endangered amphibian species in the face of parametric uncertainty. *Ecol. Model.* **220**, 148–159 (2009).

[37] Sukumaran, J. & Knowles, L. L. Multispecies coalescent delimits structure, not species. *Proc. Natl. Acad. Sci. U.S.A.* **114**, 1607–1612 (2017). 28137871 10.1073/pnas.1607921114 PMC5320999

[38] Green, D. M. & Parent, C. Variable and asymmetric introgression in a hybrid zone in the toads, *Bufo americanus* and *Bufo fowleri*. *Copeia* **2003**, 34–43 (2003).

[39] Hillis, D. M., Hillis, A. M. & Martin, R. F. Reproductive ecology and hybridization of the endangered Houston toad (*Bufo houstonensis*). *J. Herpetol.* **18**, 56–72 (1984).

[40] Milko, L. V. Integrating museum and GIS data to identify changes in species distributions driven by a disturbance-induced invasion. *Copeia* **2012**, 307–320 (2012).

[41] Valles, D. *et al.**Houston Toad Recovery: A Comprehensive Monitoring, Evaluation, and Support Program for Head-Start Success* (Texas Parks and Wildlife Department, 2019).

[42] Peterson, B. K., Weber, J. N., Kay, E. H., Fisher, H. S. & Hoekstra, H. E. Double digest RADseq: An inexpensive method for de novo SNP discovery and genotyping in model and non-model species. *PLoS ONE* **7**, e37135 (2012). 22675423 10.1371/journal.pone.0037135 PMC3365034

[43] Eaton, D. A. & Overcast, I. Ipyrad: Interactive assembly and analysis of RADseq datasets. *Bioinformatics* **36**, 2592–2594 (2020). 31904816 10.1093/bioinformatics/btz966

[44] Rognes, T., Flouri, T., Nichols, B., Quince, C. & Mahé, F. VSEARCH: A versatile open source tool for metagenomics. *PeerJ* **4**, e2584. 10.7717/peerj.2584 (2016). 27781170 10.7717/peerj.2584 PMC5075697

[45] Edgar, R. C. MUSCLE: Multiple sequence alignment with high accuracy and high throughput. *Nucleic Acids Res.* **32**, 1792–1797 (2004). 15034147 10.1093/nar/gkh340 PMC390337

[46] Eaton, D. A. PyRAD: Assembly of de novo RADseq loci for phylogenetic analyses. *Bioinformatics* **30**, 1844–1849 (2014). 24603985 10.1093/bioinformatics/btu121

[47] McCartney-Melstad, E., Gidiş, M. & Shaffer, H. B. An empirical pipeline for choosing the optimal clustering threshold in RADseq studies. *Mol. Ecol. Resour.* **19**, 1195–1204 (2019). 31058458 10.1111/1755-0998.13029

[48] De Medeiros, B. A. S. & Farrell, B. D. Whole-genome amplification in double-digest RADseq results in adequate libraries but fewer sequenced loci. *PeerJ* **6**, e5089. 10.7717/peerj.5089 (2018). 30038852 10.7717/peerj.5089 PMC6054070

[49] de Medeiros, B. A. *Matrix Condenser v.1.0*. https://github.com/brunoasm/matrix_condenser/ (2019).

[50] Darriba, D. *et al.* ModelTest-NG: A new and scalable tool for the selection of DNA and protein evolutionary models. *Mol. Biol. Evol.* **37**, 291–294 (2020). 31432070 10.1093/molbev/msz189 PMC6984357

[51] Ronquist, F. *et al.* MrBayes 3.2: Efficient Bayesian phylogenetic inference and model choice across a large model space. *Syst. Biol.* **61**, 539–542 (2012). 22357727 10.1093/sysbio/sys029 PMC3329765

[52] Langmead, B. & Salzberg, S. L. Fast gapped-read alignment with Bowtie 2. *Nat. Methods* **9**, 357–359 (2012). 22388286 10.1038/nmeth.1923 PMC3322381

[53] Streicher, J. W. The genome sequence of the common toad, *Bufo bufo* (Linnaeus, 1758). *Wellcome Open Res.* **6**, 1. 10.12688/wellcomeopenres.17298.1 (2021). 35028424 10.12688/wellcomeopenres.17298.1 PMC8729185

[54] Li, H. *et al.* The sequence alignment/map format and SAMtools. *Bioinformatics* **25**, 2078–2079 (2009). 19505943 10.1093/bioinformatics/btp352 PMC2723002

[55] Skotte, L., Korneliussen, T. S. & Albrechtsen, A. Estimating individual admixture proportions from next generation sequencing data. *Genetics* **195**, 693–702 (2013). 24026093 10.1534/genetics.113.154138 PMC3813857

[56] Waples, R. K., Albrechtsen, A. & Moltke, I. Allele frequency‐free inference of close familial relationships from genotypes or low‐depth sequencing data. *Mol. Ecol.* **28**, 35–48 (2019). 30462358 10.1111/mec.14954 PMC6850436

[57] Fumagalli, M., Vieira, F. G., Linderoth, T. & Nielsen, R. ngsTools: Methods for population genetics analyses from next-generation sequencing data. *Bioinformatics* **30**, 1486–1487 (2014). 24458950 10.1093/bioinformatics/btu041 PMC4016704

[58] Evanno, G., Regnaut, S. & Goudet, J. Detecting the number of clusters of individuals using the software STRUCTURE: A simulation study. *Mol. Ecol.* **14**, 2611–2620 (2005). 15969739 10.1111/j.1365-294X.2005.02553.x

[59] Soraggi, S., Wiuf, C. & Albrechtsen, A. Powerful inference with the D-statistic on low-coverage whole-genome data. *G3 Genes Genom. Genet.* **8**, 551–566 (2018). 10.1534/g3.117.300192 PMC5919751 29196497

