# Supplementary material for: Using genome-wide data to ascertain taxonomic status and assess population genetic structure for Houston toads (Bufo [= Anaxyrus] houstonensis)
Source: Sci Rep. 2024 Feb 8;14:3306. doi: 10.1038/s41598-024-53705-w (PMC10853240; doi:10.1038/s41598-024-53705-w)
Supplement: Supplementary file 1 — Supplementary Information. [file 41598_2024_53705_MOESM1_ESM.pdf]

Supplemental Information for:

Using genome-wide data to ascertain taxonomic status and assess population genetic structure for Houston toads (*Bufo* [=*Anaxyrus*] *houstonensis*)

Shashwat Sirsi, David Rodriguez, and Michael R.J. Forstner

Table of Contents:

**Table S1**	Page 1
**Fig. S1**	Page 5
**Fig. S2**	Page 6
**Fig. S3**	Page 7
**Fig. S4**	Page 8
**Fig. S5**	Page 9
**Fig. S6**	Page 10
**Fig. S7**	Page 11
**Fig. S8**	Page 12
**Fig. S9**	Page 13
**Fig. S10**	Page 14
**Fig. S11**	Page 15
**Fig. S12**	Page 16
**Fig. S13**	Page 17
**Fig. S14**	Page 18
**Fig. S15**	Page 19
**Fig. S16**	Page 20
**Fig. S17**	Page 21
**Fig. S18**	Page 22
**Fig. S19**	Page 23

Table S1: List of 93 toads with sample ID and field-based species-level identification with state, and county of origin in the United States. We included 48 *Bufo* [=*Anaxyrus*] *houstonensis* sampled from 2001-2015, 8 samples of *B*. *americanus* collected from 1996-2003, which included six samples of *B*. *americanus charlesmithii* and two samples of *B*. *americanus americanus*. We included 18 *B*. *woodhousii* sampled across much of their range in Texas, USA from 2002-2015 and 19 *B*. *nebulifer* sampled in Texas, USA and Tamaulipas, Mexico from 2002-2014. We provide number of raw reads per sample and have highlighted two individual samples that were excluded due to low coverage ($n < 10,000$ reads). We also indicate whether a sample was previously used in McHenry's (2010) assessment of phylogenetic status and population genetic structure in *B. houstonensis*. Since we sought comparison with McHenry's (2010) study, 40% of our *B. houstonensis* samples were also used in her study.

Sample ID	Species	Date of Collection	Country	State	County	Number of Raw Reads	In McHenry, 2010
MF1103	*Bufo a. americanus*	9/1/1996	United States	New York	Orange	6618	Yes
MF2968	*Bufo a. americanus*	10/1/1996	United States	New York	Otsego	85083	Yes
MF7399	*Bufo a. charlesmithii*	4/16/2002	United States	Oklahoma	Cleveland	6734	Yes
MF8153	*Bufo a. charlesmithii*	5/1/2003	United States	Missouri	Taney	111512	Yes
MF8154	*Bufo a. charlesmithii*	5/1/2003	United States	Missouri	Taney	124778	Yes
MF8155	*Bufo a. charlesmithii*	5/1/2003	United States	Missouri	Taney	80290	Yes
MF8156	*Bufo a. charlesmithii*	5/1/2003	United States	Missouri	Taney	160645	Yes
MF8157	*Bufo a. charlesmithii*	5/1/2003	United States	Missouri	Taney	114353	Yes
MF17311	*Bufo houstonensis*	2/14/2005	United States	Texas	Bastrop	241748	Yes
MF20012	*Bufo houstonensis*	3/30/2006	United States	Texas	Bastrop	132053	Yes
MF22259	*Bufo houstonensis*	2/20/2007	United States	Texas	Bastrop	185340	Yes
MF22324	*Bufo houstonensis*	2/27/2007	United States	Texas	Bastrop	214366	Yes
MF22325	*Bufo houstonensis*	2/27/2007	United States	Texas	Bastrop	211385	Yes
MF22338	*Bufo houstonensis*	2/28/2007	United States	Texas	Bastrop	242830	Yes
MF22397	*Bufo houstonensis*	3/12/2007	United States	Texas	Milam	201573	Yes
MF22529	*Bufo houstonensis*	4/3/2007	United States	Texas	Milam	45928	Yes
MF26270	*Bufo houstonensis*	4/2/2008	United States	Texas	Austin	155398	No
MF28150	*Bufo houstonensis*	5/16/2010	United States	Texas	Austin	126995	No
MF28151	*Bufo houstonensis*	9/16/2009	United States	Texas	Austin	199665	No
MF28152	*Bufo houstonensis*	5/16/2010	United States	Texas	Austin	69031	No
MF28154	*Bufo houstonensis*	5/29/2010	United States	Texas	Austin	63594	No
MF28655	*Bufo houstonensis*	3/5/2010	United States	Texas	Bastrop	116467	No
MF29291	*Bufo houstonensis*	5/20/2010	United States	Texas	Leon	72074	No
MF29308	*Bufo houstonensis*	5/20/2010	United States	Texas	Leon	136311	No
MF29482	*Bufo houstonensis*	5/16/2010	United States	Texas	Austin	85682	No
MF29487	*Bufo houstonensis*	9/16/2009	United States	Texas	Austin	64538	No
MF30242	*Bufo houstonensis*	3/26/2011	United States	Texas	Bastrop	116681	No
MF30243	*Bufo houstonensis*	3/26/2011	United States	Texas	Bastrop	37669	No
MF30671	*Bufo houstonensis*	5/1/2011	United States	Texas	Leon	139986	No
MF30672	*Bufo houstonensis*	5/1/2011	United States	Texas	Leon	32967	No

MF32411	*Bufo houstonensis*	8/29/2012	United States	Texas	Bastrop	102440	No
MF32412	*Bufo houstonensis*	8/10/2012	United States	Texas	Bastrop	66787	No
MF32454	*Bufo houstonensis*	6/18/2012	United States	Texas	Bastrop	44890	No
MF32712	*Bufo houstonensis*	5/16/2012	United States	Texas	Bastrop	66068	No
MF33137	*Bufo houstonensis*	1/25/2013	United States	Texas	Bastrop	56591	No
MF34592	*Bufo houstonensis*	4/16/2013	United States	Texas	Leon	257418	No
MF35945	*Bufo houstonensis*	3/31/2014	United States	Texas	Austin	64242	No
MF3618	*Bufo houstonensis*	2/13/2001	United States	Texas	Bastrop	98749	Yes
MF36351	*Bufo houstonensis*	5/29/2014	United States	Texas	Robertson	60359	No
MF37478	*Bufo houstonensis*	3/18/2015	United States	Texas	Bastrop	111332	No
MF5718	*Bufo houstonensis*	4/17/2002	United States	Texas	Lee	102013	No
MF5719	*Bufo houstonensis*	4/17/2002	United States	Texas	Lee	15657	No
MF5720	*Bufo houstonensis*	4/17/2002	United States	Texas	Lee	176382	Yes
MF5721	*Bufo houstonensis*	4/17/2002	United States	Texas	Lee	99321	No
MF5722	*Bufo houstonensis*	4/17/2002	United States	Texas	Lee	75735	No
MF5723	*Bufo houstonensis*	4/17/2002	United States	Texas	Lee	257415	No
MF5724	*Bufo houstonensis*	4/17/2002	United States	Texas	Lee	80864	No
MF5727	*Bufo houstonensis*	4/17/2002	United States	Texas	Lee	140870	Yes
MF5728	*Bufo houstonensis*	4/17/2002	United States	Texas	Lee	260245	Yes
MF5729	*Bufo houstonensis*	4/17/2002	United States	Texas	Lee	95588	Yes
MF5756	*Bufo houstonensis*	4/17/2002	United States	Texas	Lee	226900	Yes
MF5757	*Bufo houstonensis*	4/17/2002	United States	Texas	Lee	354148	Yes
MF5758	*Bufo houstonensis*	4/17/2002	United States	Texas	Lee	214954	Yes
MF5759	*Bufo houstonensis*	4/17/2002	United States	Texas	Lee	28232	Yes
MF5760	*Bufo houstonensis*	4/17/2002	United States	Texas	Lee	69151	Yes
MF5761	*Bufo houstonensis*	4/17/2002	United States	Texas	Lee	55919	Yes
MF16911	*Bufo nebulifer*	10/23/2004	Mexico	Tamaulipas		149899	Yes
MF20074	*Bufo nebulifer*	3/30/2006	United States	Texas	Leon	302237	Yes
MF20792	*Bufo nebulifer*	6/18/2006	United States	Texas	Burleson	298621	Yes
MF20794	*Bufo nebulifer*	6/18/2006	United States	Texas	Lavaca	97024	Yes
MF20796	*Bufo nebulifer*	6/18/2006	United States	Texas	Burleson	102897	Yes
MF20960	*Bufo nebulifer*	6/23/2006	United States	Texas	Aransas	180086	Yes
MF22063	*Bufo nebulifer*	8/7/2002	United States	Texas	Cameron	128722	Yes
MF22434	*Bufo nebulifer*	3/27/2007	United States	Texas	Bastrop	216579	Yes
MF22435	*Bufo nebulifer*	3/27/2007	United States	Texas	Bastrop	185026	Yes
MF22487	*Bufo nebulifer*	3/29/2007	United States	Texas	Bastrop	194258	Yes
MF23406	*Bufo nebulifer*	7/28/2007	United States	Texas	Hill	58835	Yes
MF23407	*Bufo nebulifer*	7/28/2007	United States	Texas	Hill	55509	Yes
MF23410	*Bufo nebulifer*	7/28/2007	United States	Texas	Hill	79869	Yes

MF28535	*Bufo nebulifer*	10/5/2009	United States	Texas	Guadalupe	72310	No
MF33655	*Bufo nebulifer*	4/17/2013	United States	Texas	Colorado	123311	No
MF33658	*Bufo nebulifer*	4/17/2013	United States	Texas	Colorado	111997	No
MF34879	*Bufo nebulifer*	4/8/2012	United States	Texas	Bastrop	54476	No
MF35921	*Bufo nebulifer*	4/2/2014	United States	Texas	Bastrop	124617	No
MF9537	*Bufo nebulifer*	7/9/2004	Mexico	Tamaulipas		281656	No
MF20088	*Bufo woodhousii*	4/10/2006	United States	Texas	Hill	136433	Yes
MF20940	*Bufo woodhousii*	6/25/2006	United States	Oklahoma	Potowatomi	189968	No
MF23422	*Bufo woodhousii*	4/17/2002	United States	Texas	Hill	82948	No
MF26110	*Bufo woodhousii*	3/7/2008	United States	Texas	Henderson	106325	No
MF26140	*Bufo woodhousii*	4/8/2008	United States	Texas	Anderson	257821	No
MF26873	*Bufo woodhousii*	5/9/2008	United States	Texas	Leon	81056	No
MF26874	*Bufo woodhousii*	5/9/2008	United States	Texas	Leon	106812	No
MF27039	*Bufo woodhousii*	9/5/2008	United States	Texas	Randall	160620	No
MF27043	*Bufo woodhousii*	9/5/2008	United States	Texas	Deaf Smith	132384	No
MF27044	*Bufo woodhousii*	9/5/2008	United States	Texas	Deaf Smith	128517	No
MF27053	*Bufo woodhousii*	9/5/2008	United States	Texas	Parmer	155302	No
MF27059	*Bufo woodhousii*	9/6/2008	United States	Texas	Castro	153163	No
MF27062	*Bufo woodhousii*	9/6/2008	United States	Texas	Parmer	95998	No
MF7398	*Bufo woodhousii*	4/17/2002	United States	Oklahoma	Cleveland	37530	Yes
MFAAC1	*Bufo woodhousii*	10/1/2015	United States	Oklahoma	McCurtain	20641	No
MFAAC2	*Bufo woodhousii*	10/1/2015	United States	Oklahoma	McCurtain	31099	No
MFAAC3	*Bufo woodhousii*	10/1/2015	United States	Oklahoma	McCurtain	21101	No
MFAAC4	*Bufo woodhousii*	10/1/2015	United States	Oklahoma	McCurtain	83084	No

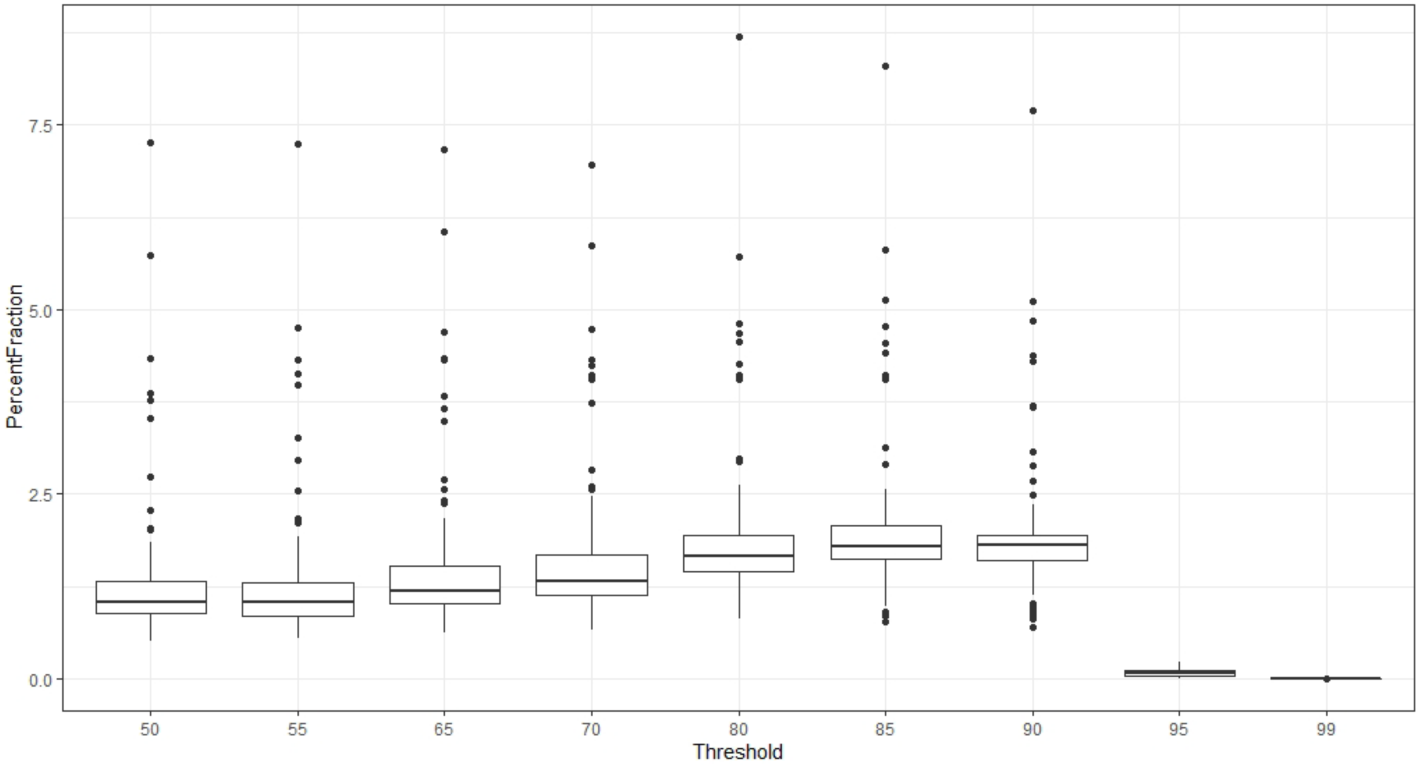

Figure S1: Percent fraction of paralogous clusters (y-axis) inferred across ten runs in ipyrad version 0.9.59, at clustering thresholds that ranged from 0.50 to 0.99 (x-axis). We used ddRAD data from 93 individuals that included 8 American Toads (*Bufo* [=*Anaxyrus*] *americanus*), 48 Houston Toads (*B*. *houstonensis*), 18 Woodhouse's Toads (*B*. *woodhousii*), and 19 Gulf Coast Toads (*B*. *nebulifer*). We observed a sharp decrease in the percentage of inferred paralogous clusters at a clustering threshold of 0.95.

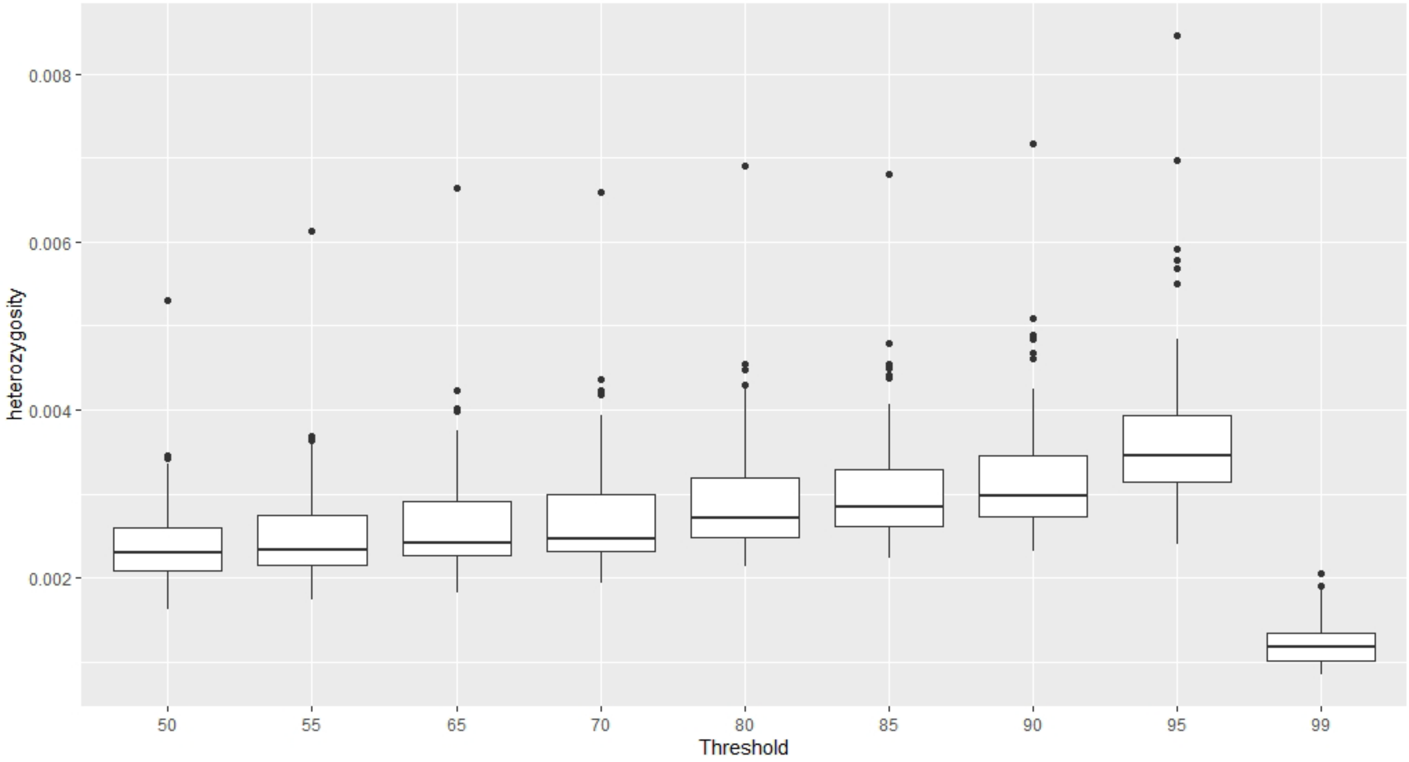

Figure S2: Individual heterozygosity (y-axis) estimated across ten runs in ipyrad version 0.9.59, at clustering thresholds that ranged from 0.50 to 0.99 (x-axis). We used ddRAD data from 93 individuals that included 8 American Toads (*Bufo* [=*Anaxyrus*] *americanus*), 48 Houston Toads (*B*. *houstonensis*), 18 Woodhouse's Toads (*B*. *woodhousii*), and 19 Gulf Coast Toads (*B*. *nebulifer*)**.** We observed highest values of individual heterozygosity at a clustering threshold of 0.95.

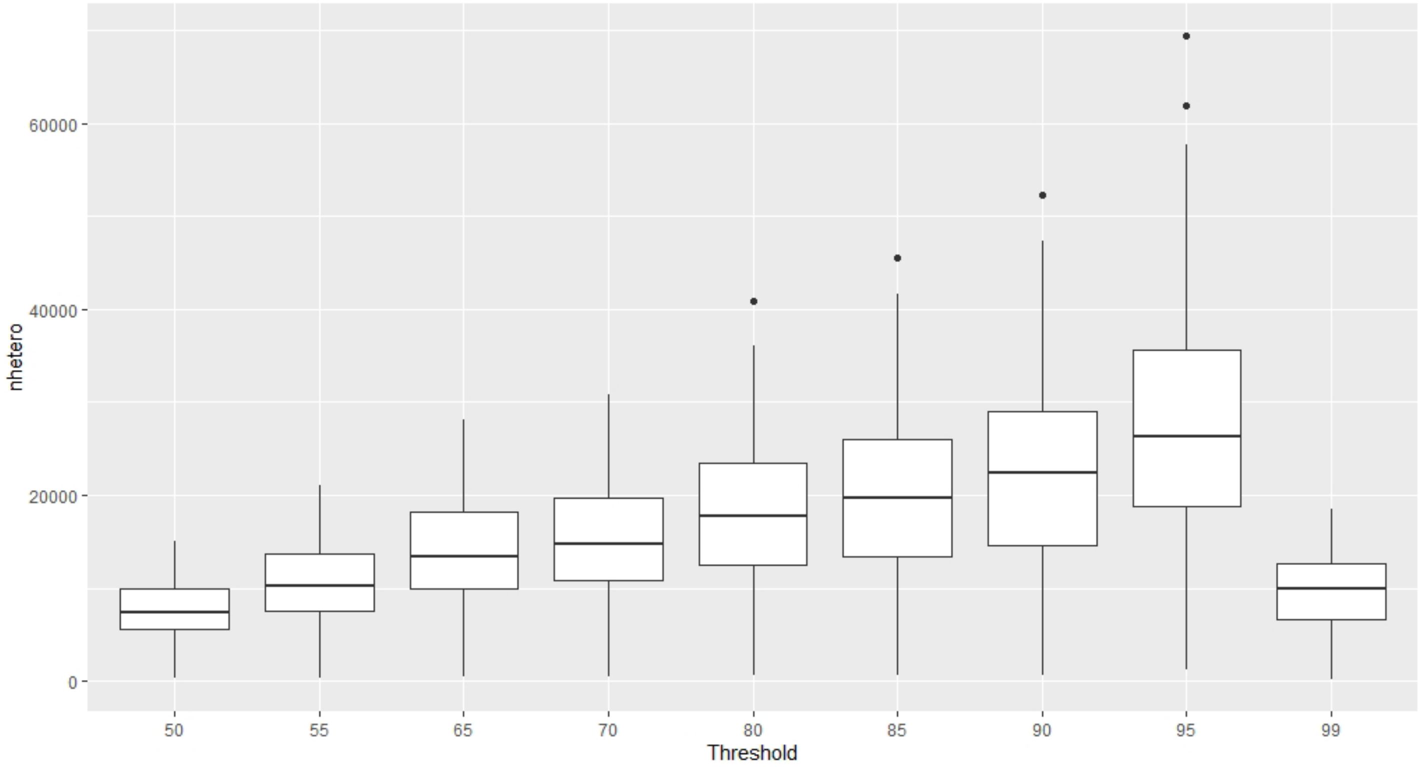

Figure S3: Total number of SNPs (y-axis) observed across ten runs in ipyrad version 0.9.59, at clustering thresholds that ranged from 0.50 to 0.99 (x-axis). We used ddRAD data from 93 individuals that included 8 American Toads (*Bufo* [=*Anaxyrus*] *americanus*), 48 Houston Toads (*B*. *houstonensis*), 18 Woodhouse's Toads (*B*. *woodhousii*), and 19 Gulf Coast Toads (*B*. *nebulifer*)**.** We observed the greatest number of SNPs at a clustering threshold of 0.95.

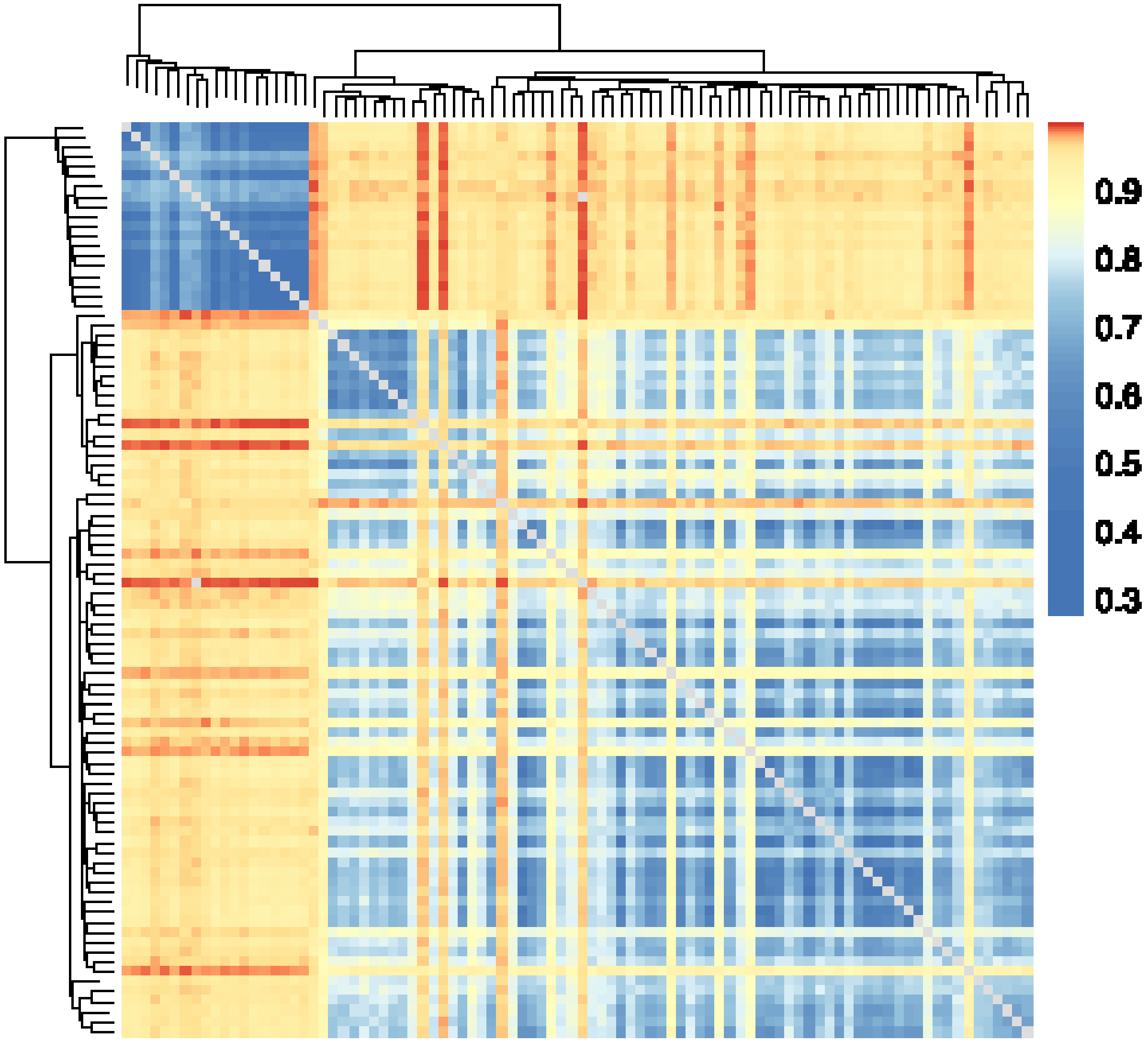

Figure S4: Distance based dendrogram of samples with a heatmap of pairwise data-missingness between samples estimated at a clustering threshold of 0.50 in ipyrad version 0.9.59. We used ddRAD data from 93 individuals that included 8 American Toads (*Bufo* [=*Anaxyrus*] *americanus*), 48 Houston Toads (*B. houstonensis*), 18 Woodhouse's Toads (*B. woodhousii*), and 19 Gulf Coast Toads (*B. nebulifer*)**.** Minimum and maximum pairwise data-missingness were 0.31 and 0.99, respectively. Pearson's Correlation Coefficient between data missingness and genetic similarity was 0.67.

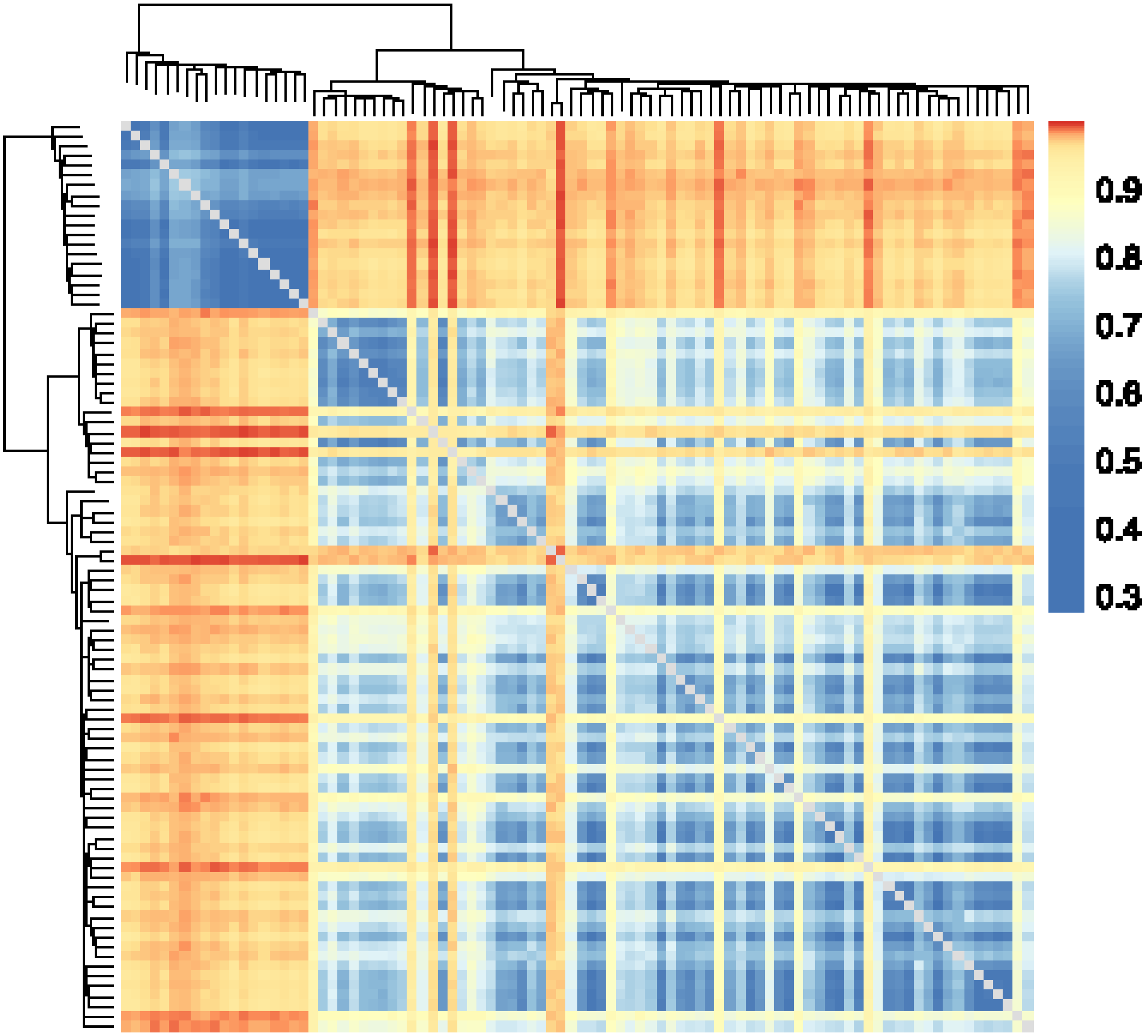

Figure S5: Distance based dendrogram of samples with a heatmap of pairwise data-missingness between samples estimated at a clustering threshold of 0.55 in ipyrad version 0.9.59. We used ddRAD data from 93 individuals that included 8 American Toads (*Bufo* [=*Anaxyrus*] *americanus*), 48 Houston Toads (*B. houstonensis*), 18 Woodhouse's Toads (*B. woodhousii*), and 19 Gulf Coast Toads (*B. nebulifer*). Minimum and maximum pairwise data-missingness were 0.28 and 0.99, respectively. Pearson's Correlation Coefficient between data missingness and genetic similarity was 0.67.

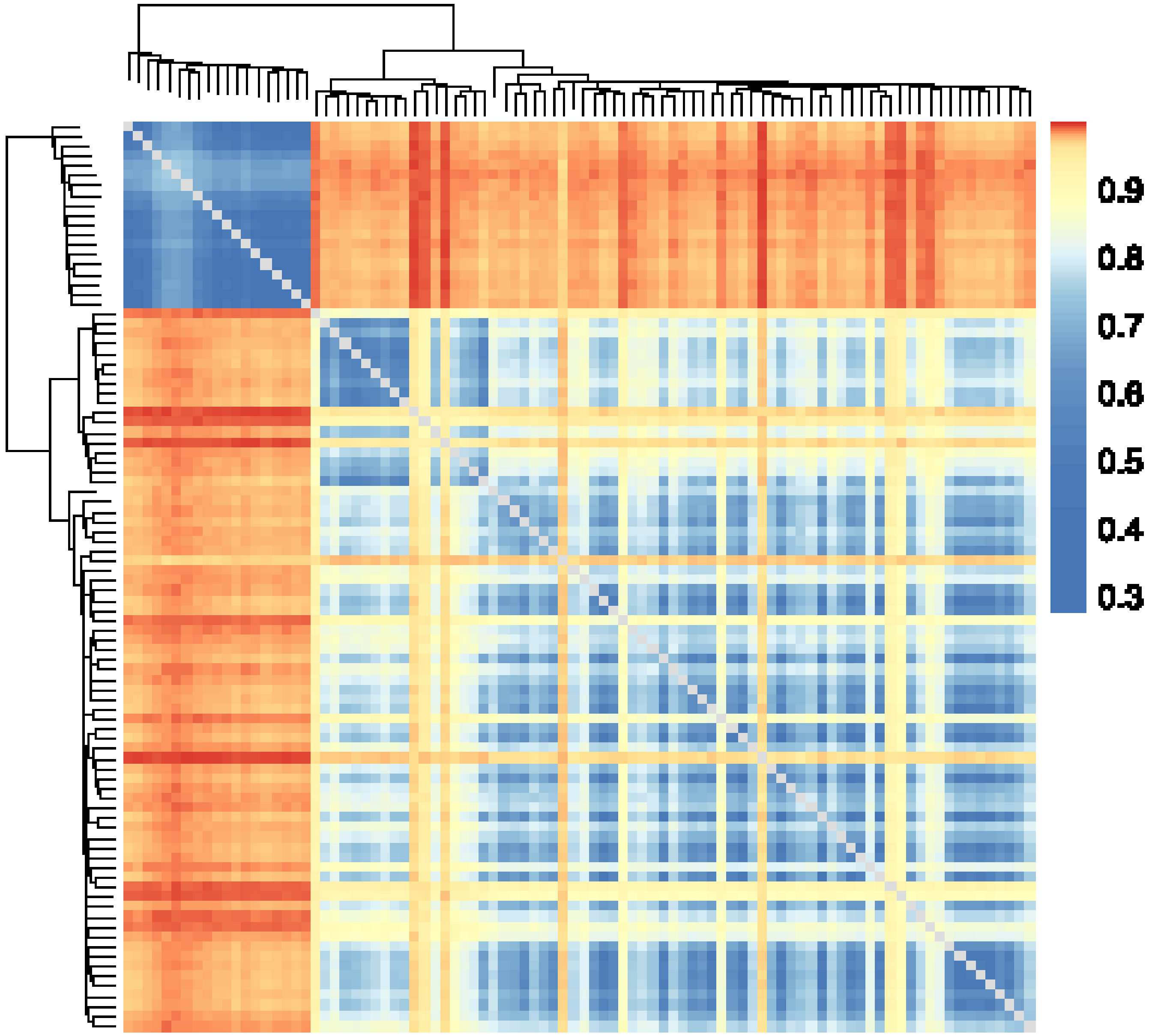

Figure S6: Distance based dendrogram of samples with a heatmap of pairwise data-missingness between samples estimated at a clustering threshold of 0.65 in ipyrad version 0.9.59. We used ddRAD data from 93 individuals that included 8 American Toads (*Bufo* [=*Anaxyrus*] *americanus*), 48 Houston Toads (*B. houstonensis*), 18 Woodhouse's Toads (*B. woodhousii*), and 19 Gulf Coast Toads (*B. nebulifer*)**.** Minimum and maximum pairwise data-missingness were 0.28 and 0.99, respectively. Pearson's Correlation Coefficient between data missingness and genetic similarity was 0.69.

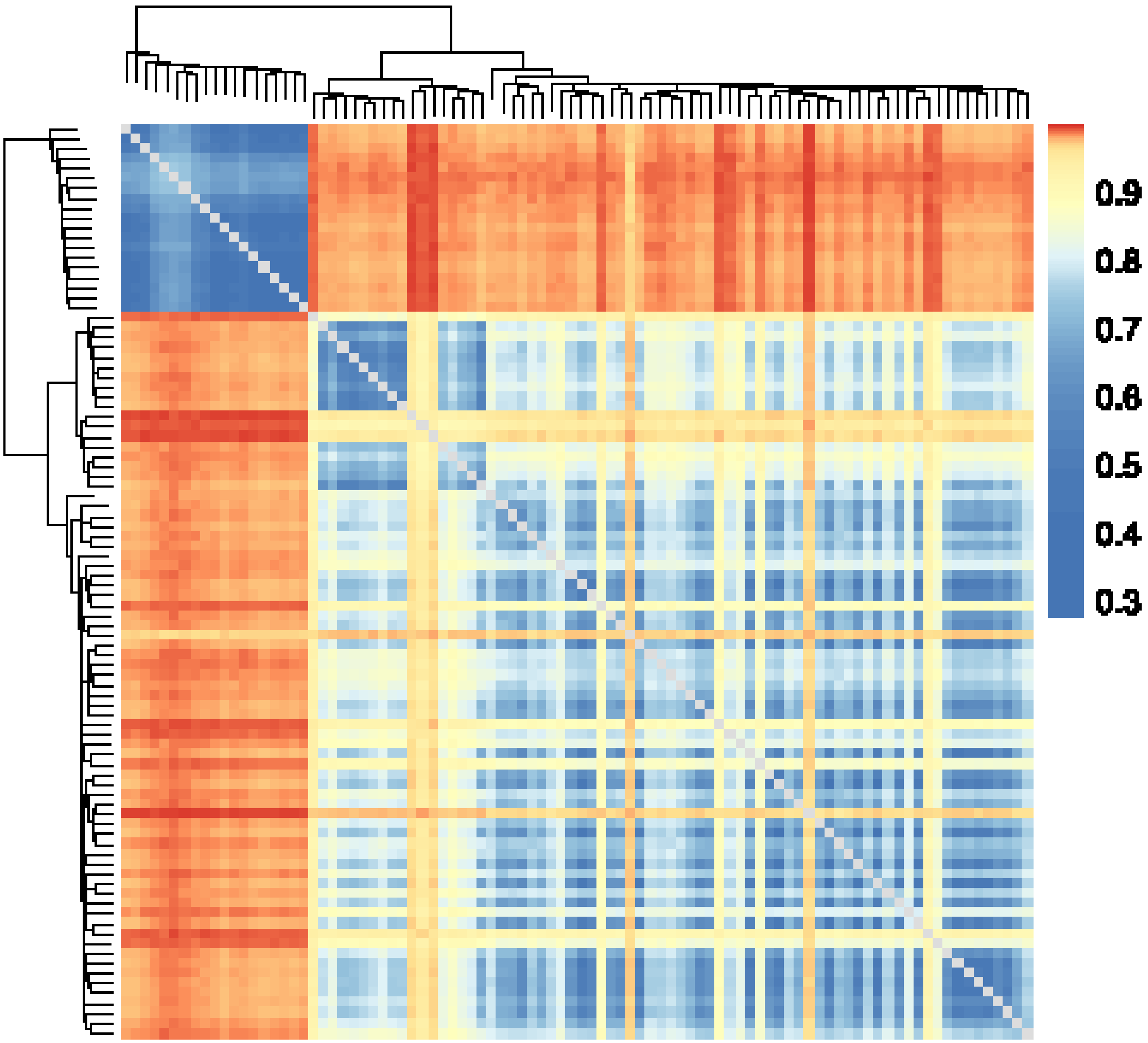

Figure S7: Distance based dendrogram of samples with a heatmap of pairwise data-missingness between samples estimated at a clustering threshold of 0.70 in ipyrad version 0.9.59. We used ddRAD data from 93 individuals that included 8 American Toads (*Bufo* [=*Anaxyrus*] *americanus*), 48 Houston Toads (*B. houstonensis*), 18 Woodhouse's Toads (*B. woodhousii*), and 19 Gulf Coast Toads (*B. nebulifer*)**.** Minimum and maximum pairwise data-missingness were 0.29 and 0.99, respectively. Pearson's Correlation Coefficient between data missingness and genetic similarity was 0.69.

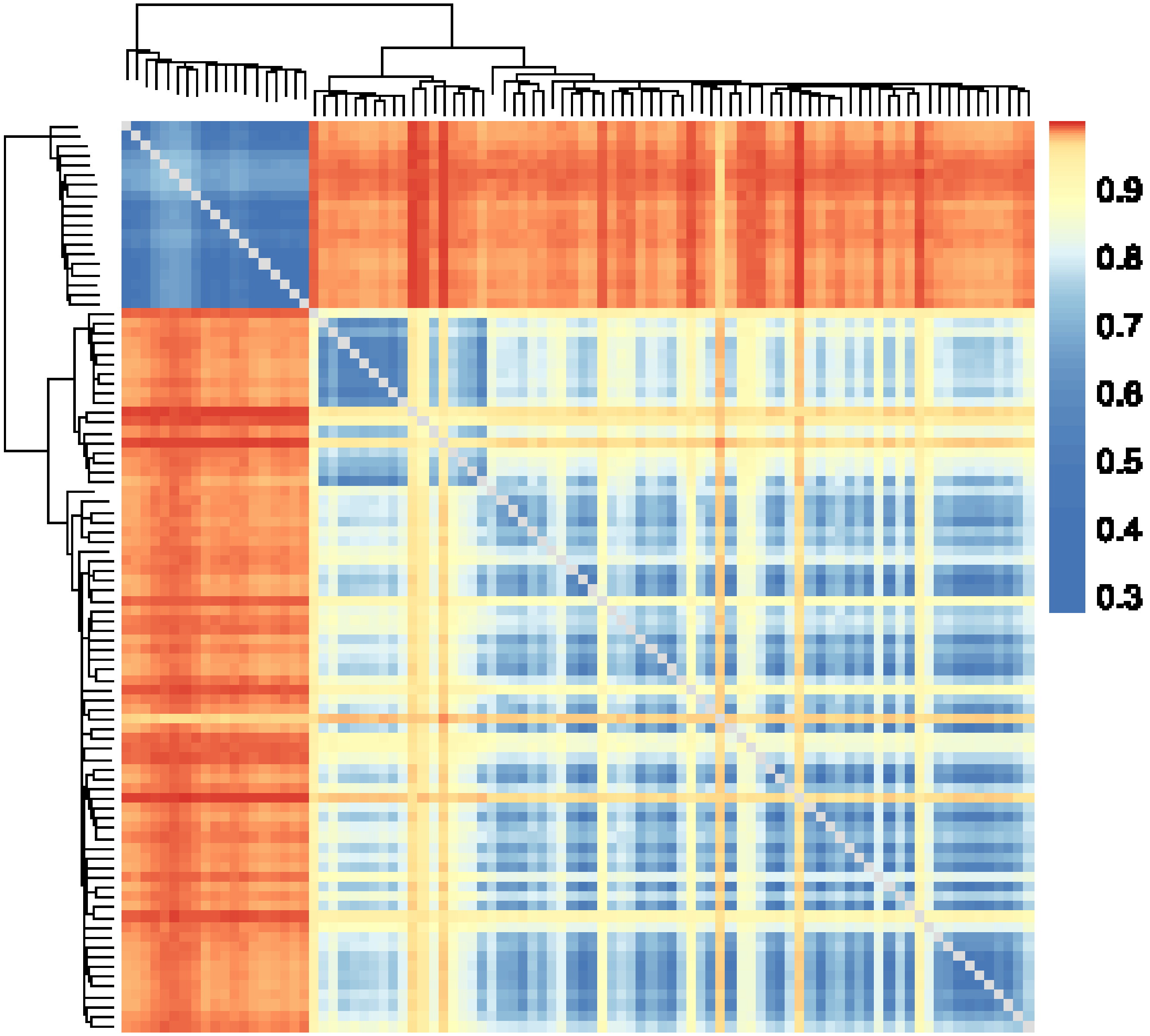

Figure S8: Distance based dendrogram of samples with a heatmap of pairwise data-missingness between samples estimated at a clustering threshold of 0.80 in ipyrad version 0.9.59. We used ddRAD data from 93 individuals that included 8 American Toads (*Bufo* [=*Anaxyrus*] *americanus*), 48 Houston Toads (*B. houstonensis*), 18 Woodhouse's Toads (*B. woodhousii*), and 19 Gulf Coast Toads (*B. nebulifer*)**.** Minimum and maximum pairwise data-missingness were 0.29 and 0.99, respectively. Pearson's Correlation Coefficient between data missingness and genetic similarity was 0.70.

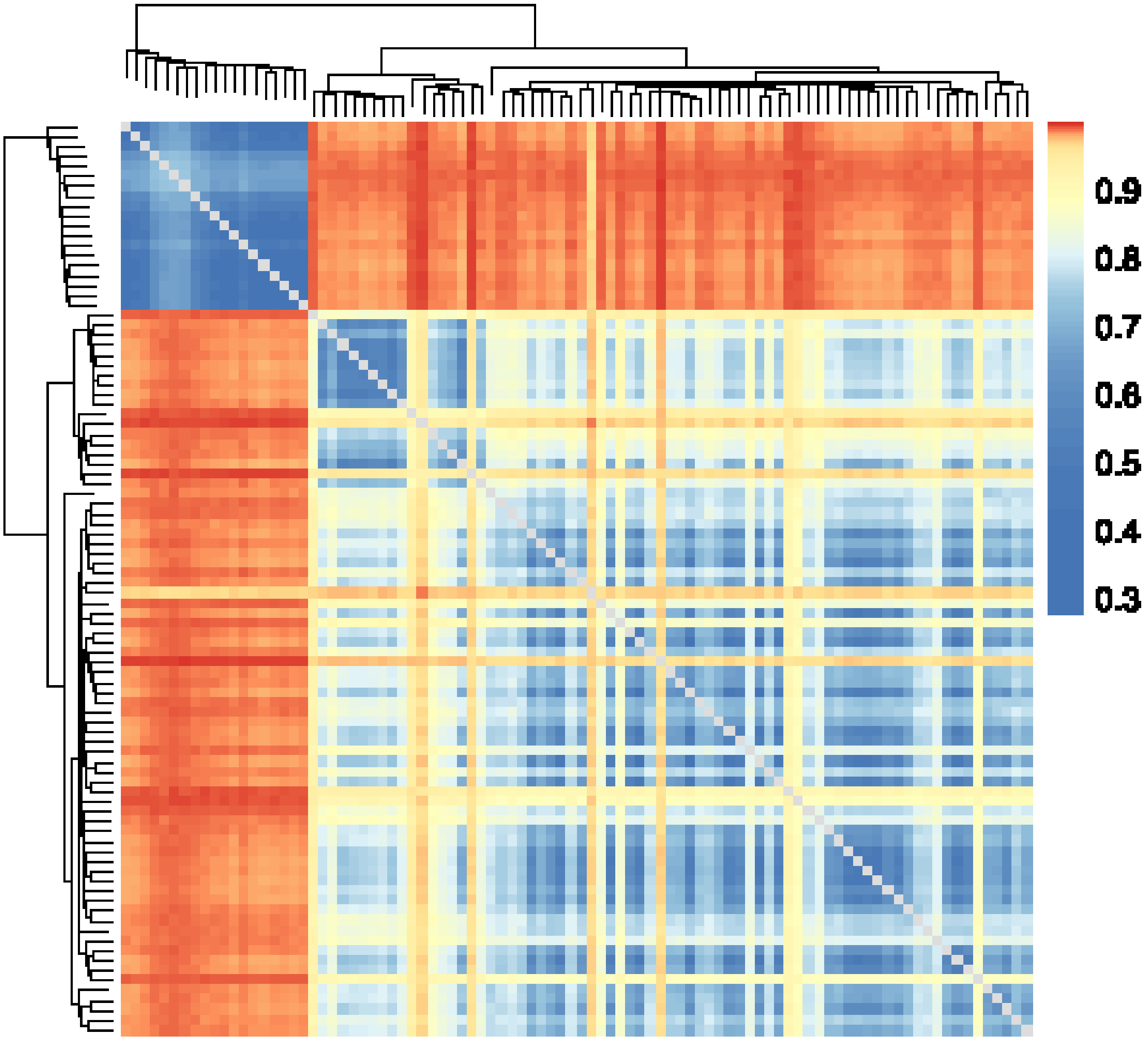

Figure S9: Distance based dendrogram of samples with a heatmap of pairwise data-missingness between samples estimated at a clustering threshold of 0.85 in ipyrad version 0.9.59. We used ddRAD data from 93 individuals that included 8 American Toads (*Bufo* [=*Anaxyrus*] *americanus*), 48 Houston Toads (*B. houstonensis*), 18 Woodhouse's Toads (*B. woodhousii*), and 19 Gulf Coast Toads (*B. nebulifer*)**.** Minimum and maximum pairwise data-missingness were 0.28 and 0.99, respectively. Pearson's Correlation Coefficient between data missingness and genetic similarity was 0.69.

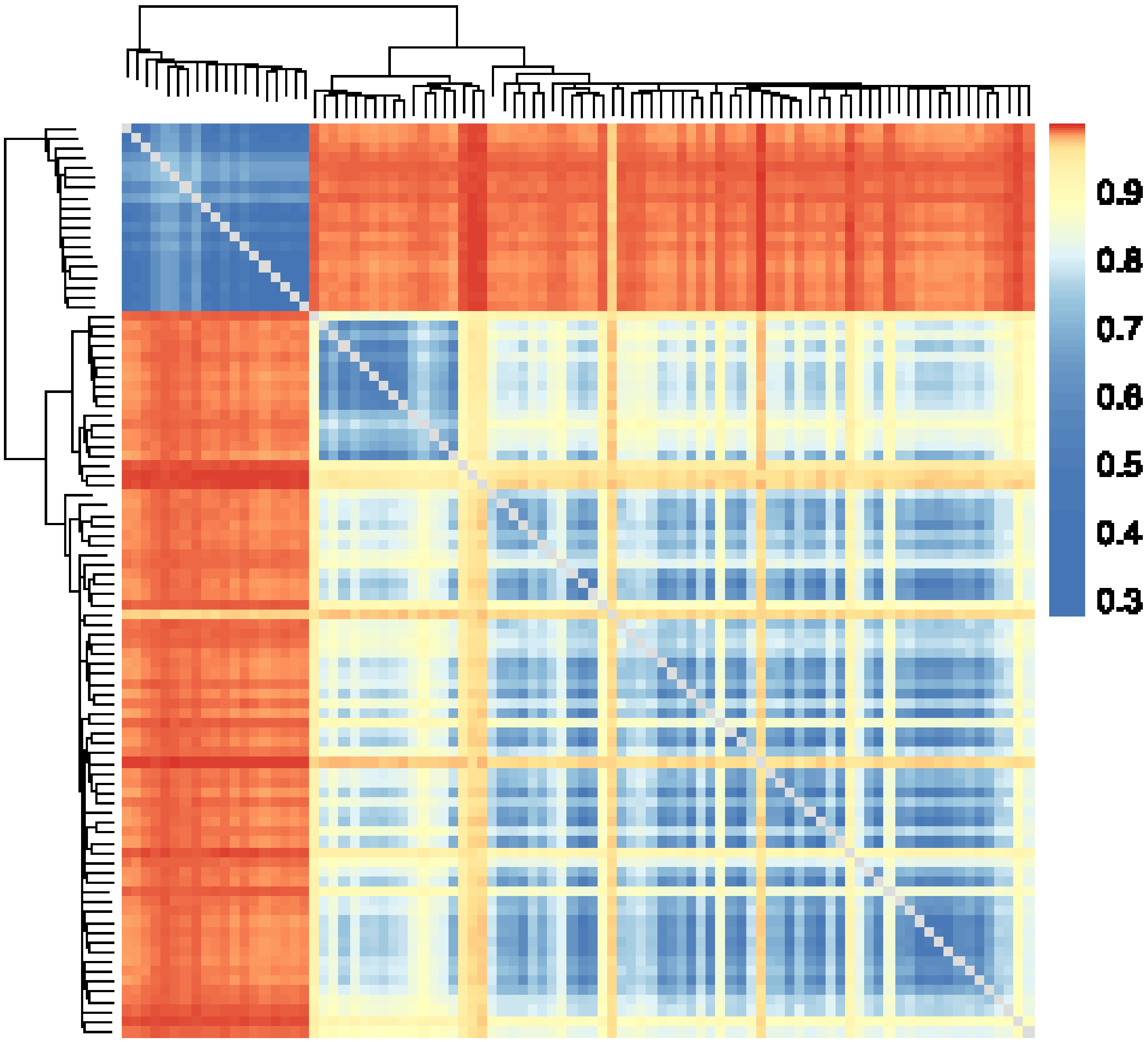

Figure S10: Distance based dendrogram of samples with a heatmap of pairwise data-missingness between samples estimated at a clustering threshold of 0.90 in ipyrad version 0.9.59. We used ddRAD data from 93 individuals that included 8 American Toads (*Bufo* [=*Anaxyrus*] *americanus*), 48 Houston Toads (*B. houstonensis*), 18 Woodhouse's Toads (*B. woodhousii*), and 19 Gulf Coast Toads (*B. nebulifer*)**.** Minimum and maximum pairwise data-missingness were 0.28 and 0.99, respectively. Pearson's Correlation Coefficient between data missingness and genetic similarity was 0.69.

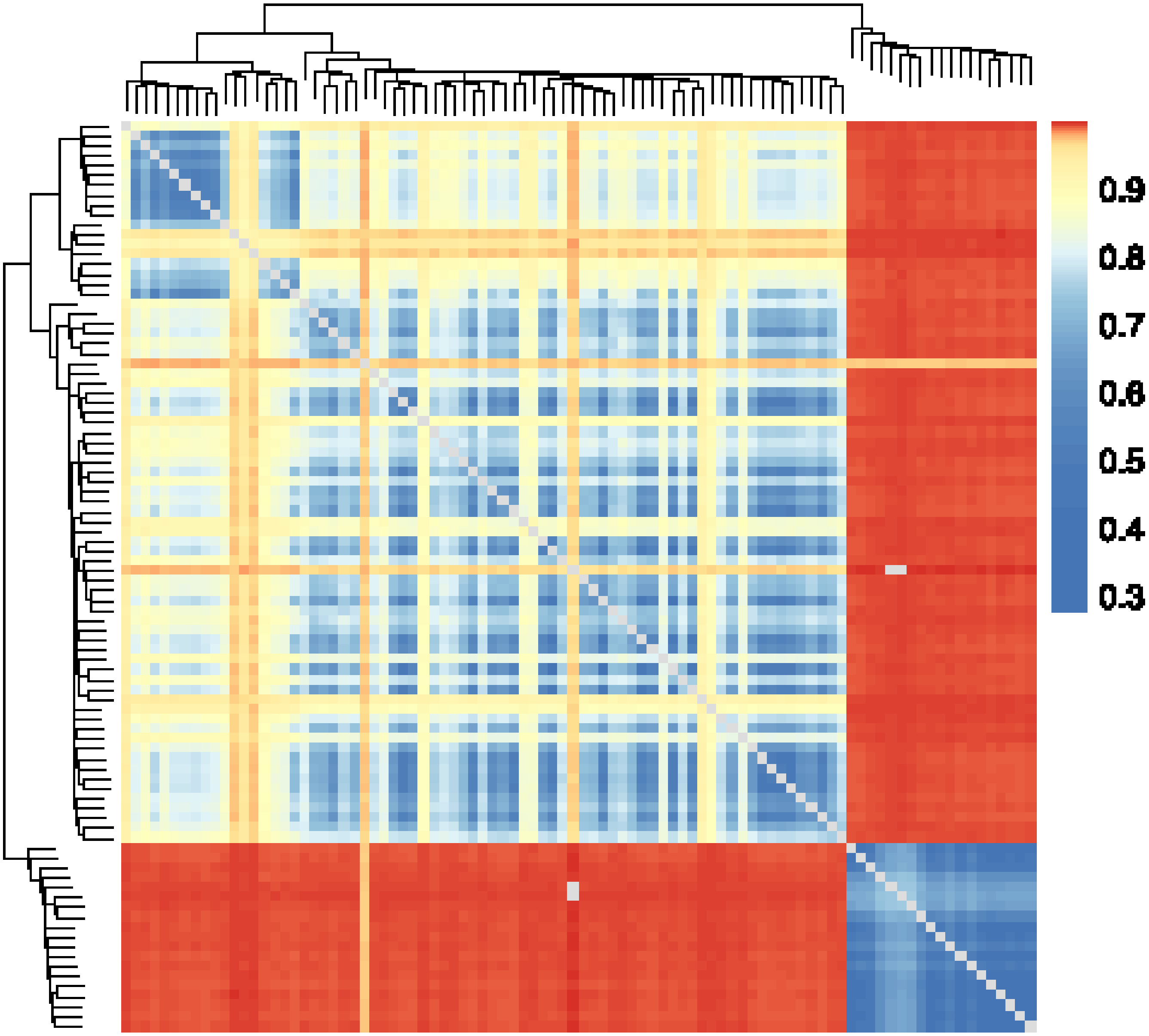

Figure S11: Distance based dendrogram of samples with a heatmap of pairwise data-missingness between samples estimated at a clustering threshold of 0.95 in ipyrad version 0.9.59. We used ddRAD data from 93 individuals that included 8 American Toads (*Bufo* [=*Anaxyrus*] *americanus*), 48 Houston Toads (*B. houstonensis*), 18 Woodhouse's Toads (*B. woodhousii*), and 19 Gulf Coast Toads (*B. nebulifer*)**.** Minimum and maximum pairwise data-missingness were 0.28 and 0.99, respectively. Pearson's Correlation Coefficient between data missingness and genetic similarity was 0.70.

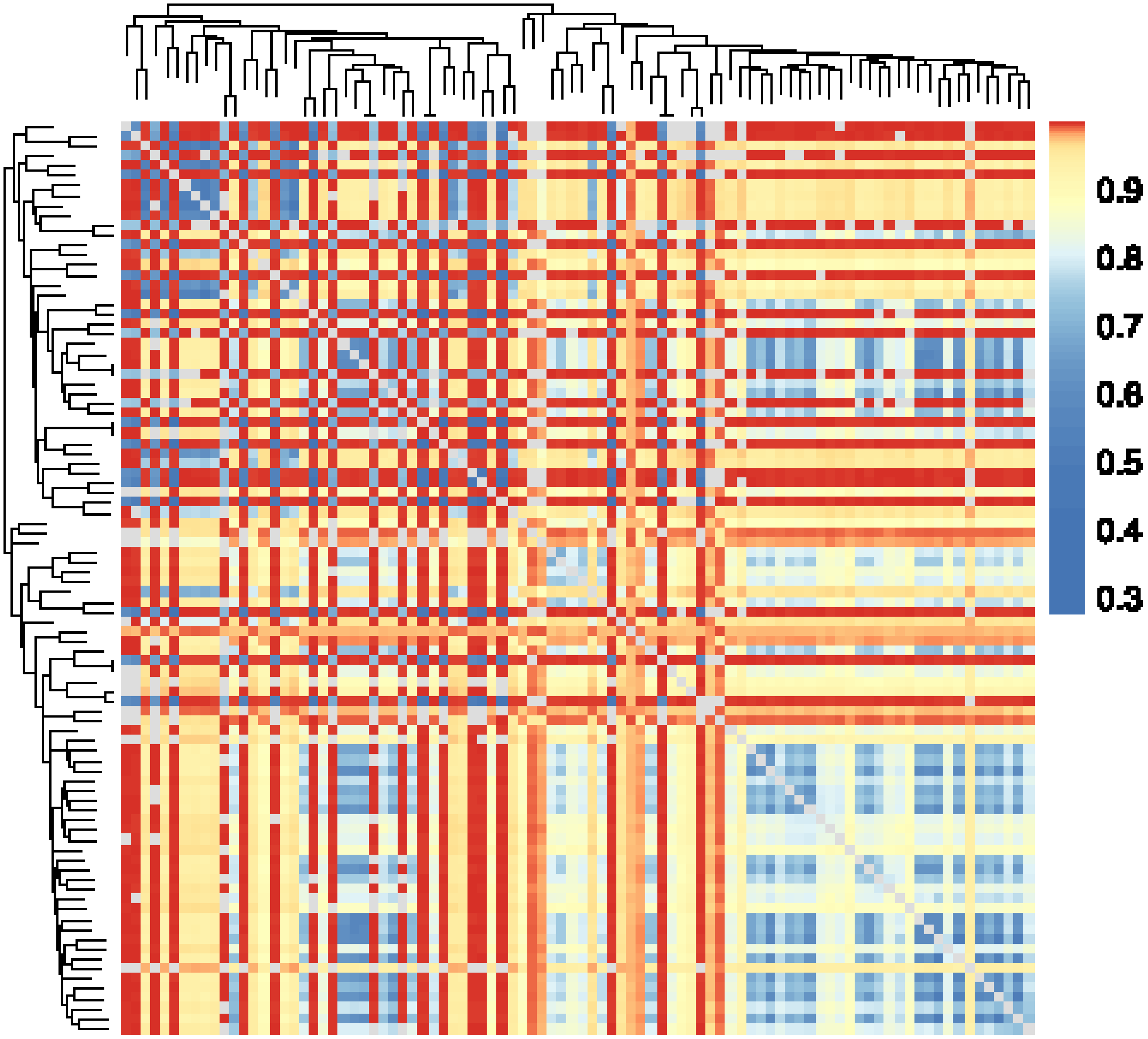

Figure S12: Distance based dendrogram of samples with a heatmap of pairwise data-missingness between samples estimated at a clustering threshold of 0.99 in ipyrad version 0.9.59. We used ddRAD data from 93 individuals that included 8 American Toads (*Bufo* [=*Anaxyrus*] *americanus*), 48 Houston Toads (*B. houstonensis*), 18 Woodhouse's Toads (*B. woodhousii*), and 19 Gulf Coast Toads (*B. nebulifer*)**.** Minimum and maximum pairwise data-missingness were 0.32 and 0.99, respectively. Pearson's Correlation Coefficient between data missingness and genetic similarity was 0.53.

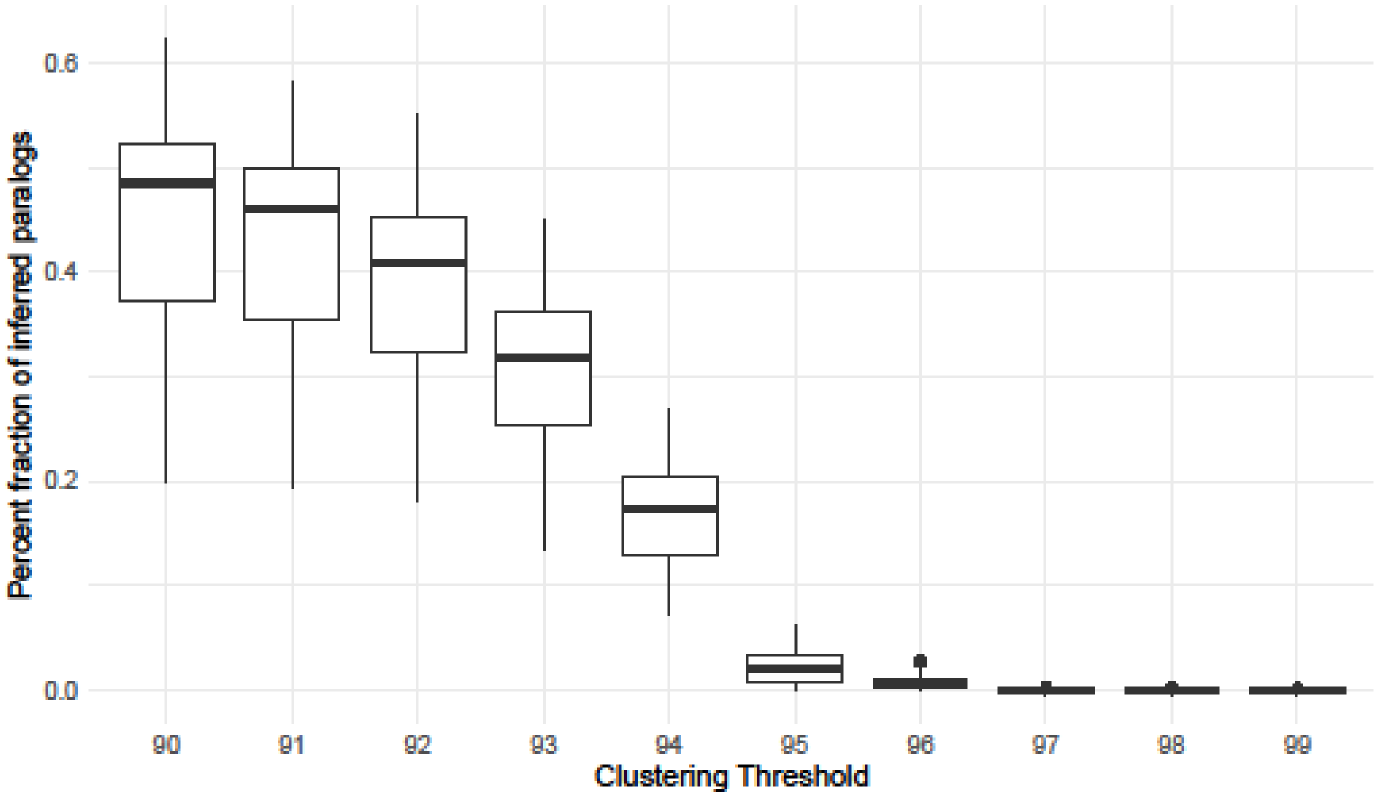

Figure S13: Percent fraction of paralogous clusters (y-axis) inferred across ten runs in ipyrad version 0.9.59, at clustering thresholds that ranged from 0.90 to 0.99 (x-axis). We used ddRAD data from 93 individuals that included 8 American Toads (*Bufo* [=*Anaxyrus*] *americanus*), 48 Houston Toads (*B*. *houstonensis*), 18 Woodhouse's Toads (*B*. *woodhousii*), and 19 Gulf Coast Toads (*B*. *nebulifer*). We observed a sharp decrease in the percentage of inferred paralogous clusters at a clustering threshold of 0.95.

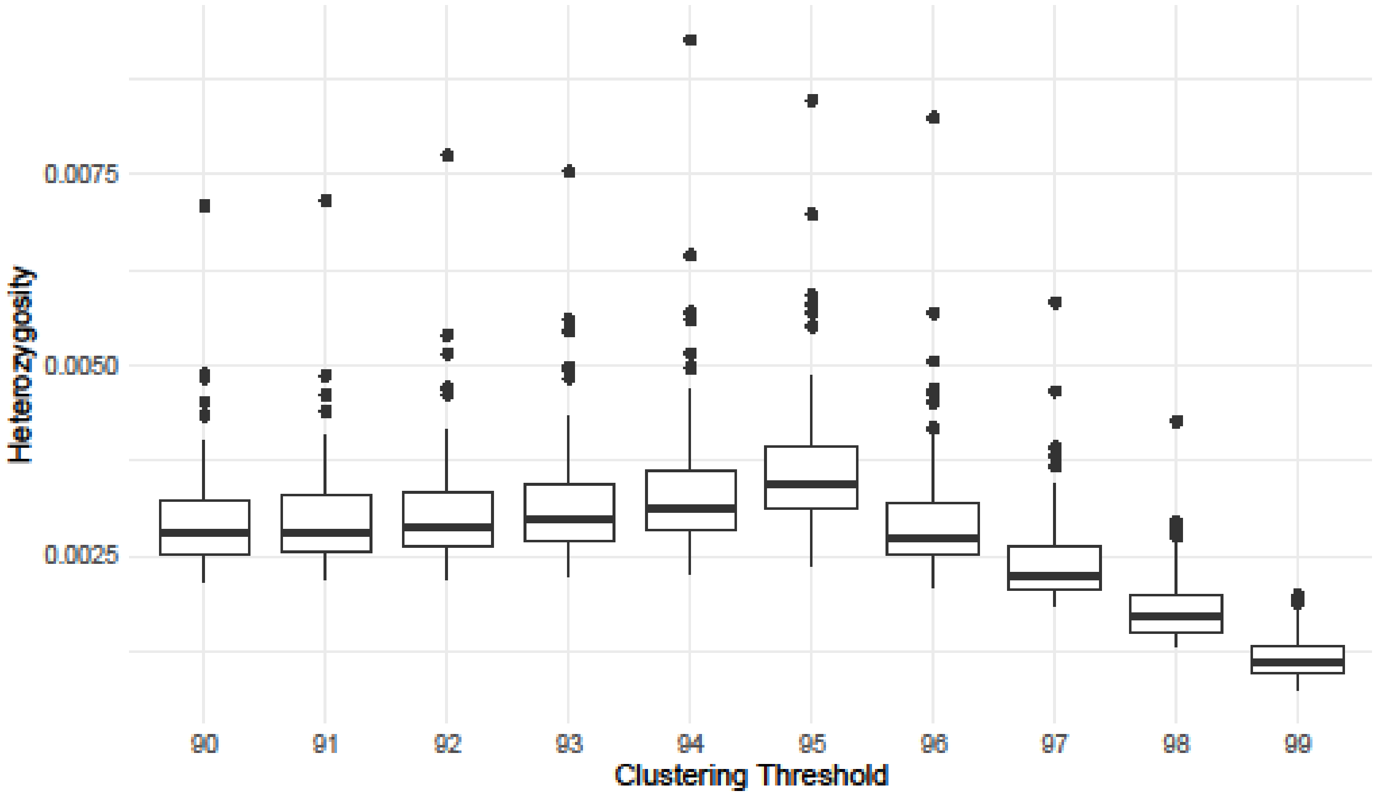

Figure S14: Individual heterozygosity (y-axis) estimated across ten runs in ipyrad version 0.9.59, at clustering thresholds that ranged from 0.90 to 0.99 (x-axis). We used ddRAD data from 93 individuals that included 8 American Toads (*Bufo* [=*Anaxyrus*] *americanus*), 48 Houston Toads (*B*. *houstonensis*), 18 Woodhouse's Toads (*B*. *woodhousii*), and 19 Gulf Coast Toads (*B*. *nebulifer*)**.** We observed highest values of individual heterozygosity at a clustering threshold of 0.95.

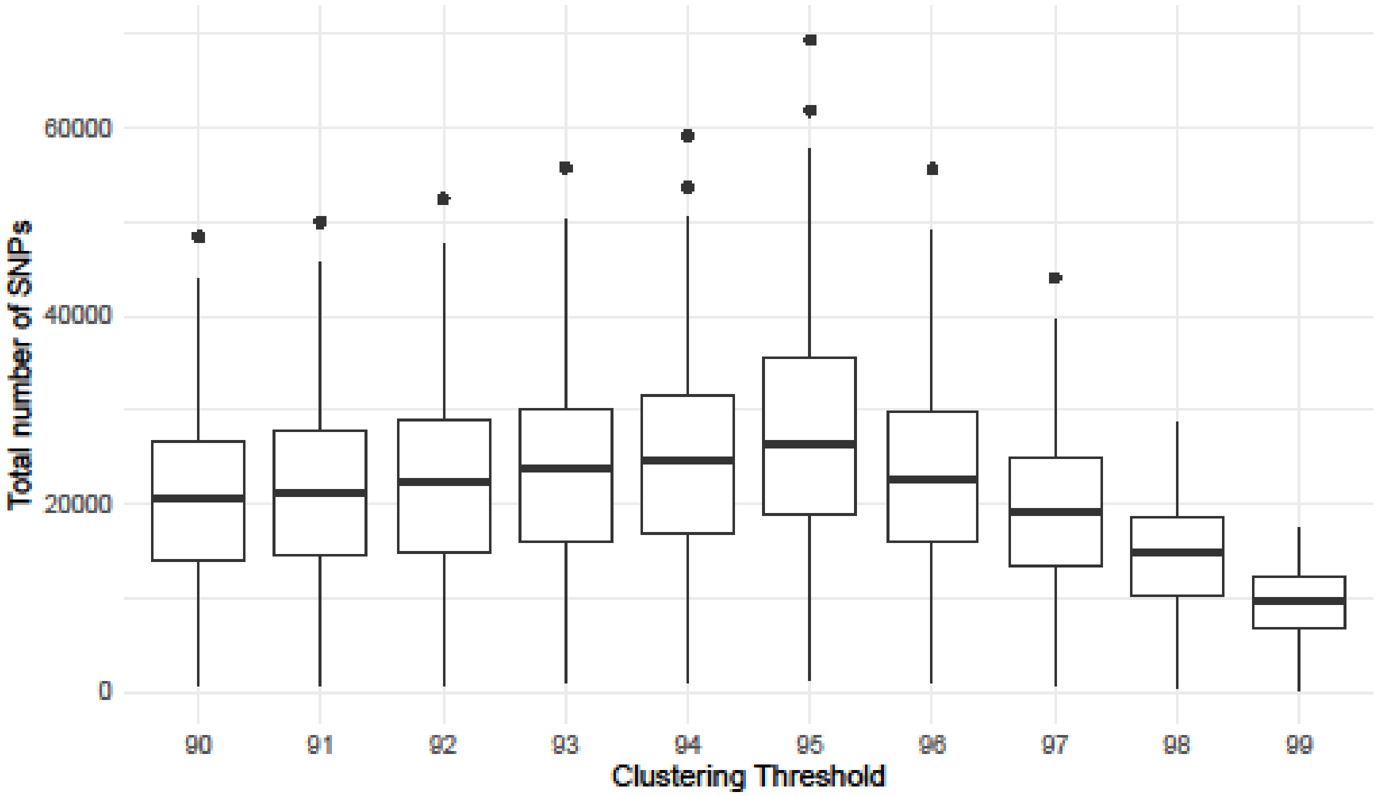

Figure S15: Total number of SNPs (y-axis) observed across ten runs in ipyrad version 0.9.59, at clustering thresholds that ranged from 0.90 to 0.99 (x-axis). We used ddRAD data from 93 individuals that included 8 American Toads (*Bufo* [=*Anaxyrus*] *americanus*), 48 Houston Toads (*B. houstonensis*), 18 Woodhouse's Toads (*B. woodhousii*), and 19 Gulf Coast Toads (*B. nebulifer*)**.** We observed the greatest number of SNPs at a clustering threshold of 0.95.

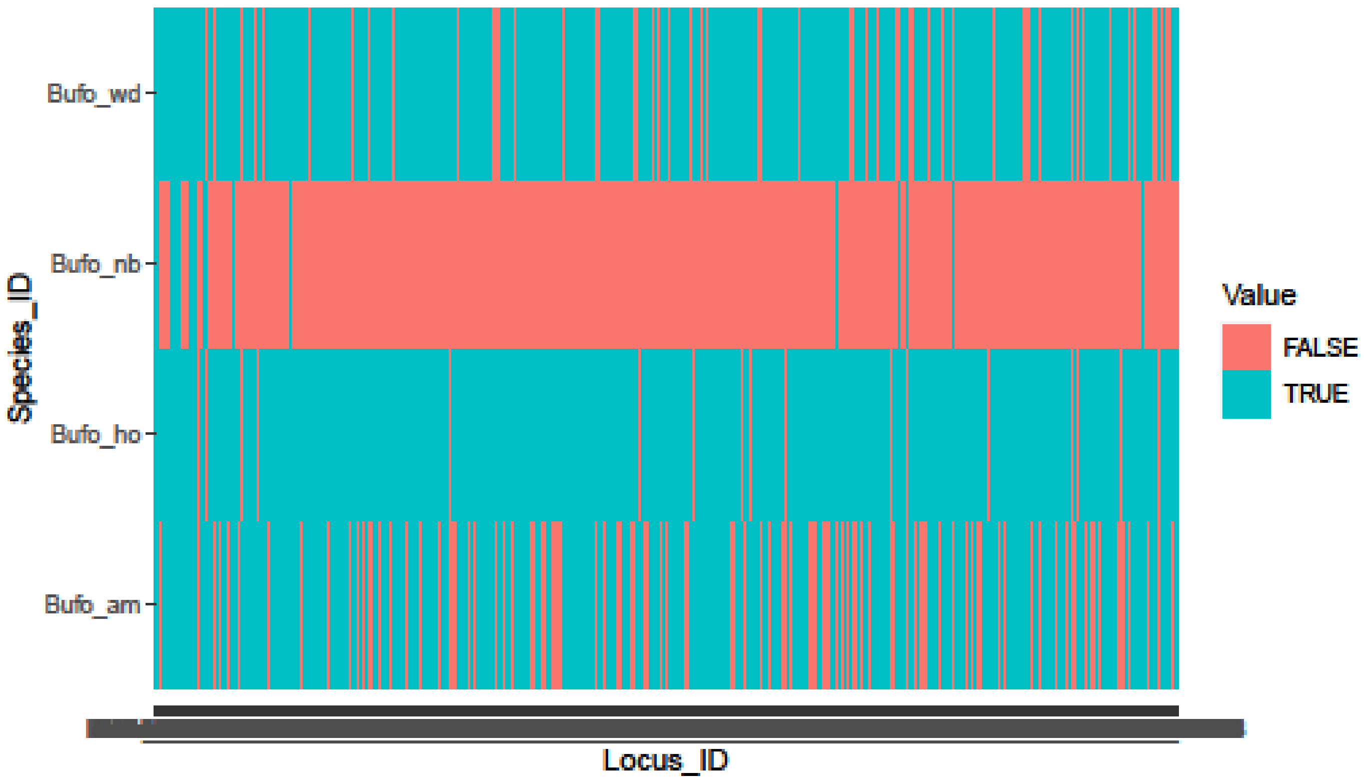

Figure S16: Occupancy matrix visualizing the distribution of data per locus across 91 individual toads in Matrix Condenser. This filtered dataset includes 6 American Toads (*Bufo* [=*Anaxyrus*] *americanus*), 48 Houston Toads (*B. houstonensis*), 18 Woodhouse's Toads (*B. woodhousii*), and 19 Gulf Coast Toads (*B. nebulifer*). This matrix excludes two *B. americanus* individuals (MF1103 and MF7399) with fewer than 10,000 reads and greater than 95% missing data. We also only retained loci that were recovered for a minimum of 41 samples, with 49.8% missing sites in our sequence matrix for these 91 samples. Despite a more stringent filtering threshold, we continued to see a systematic pattern to missing data- with few loci overlapping between Middle American (i.e., Bufo_nb) and Nearctic species.

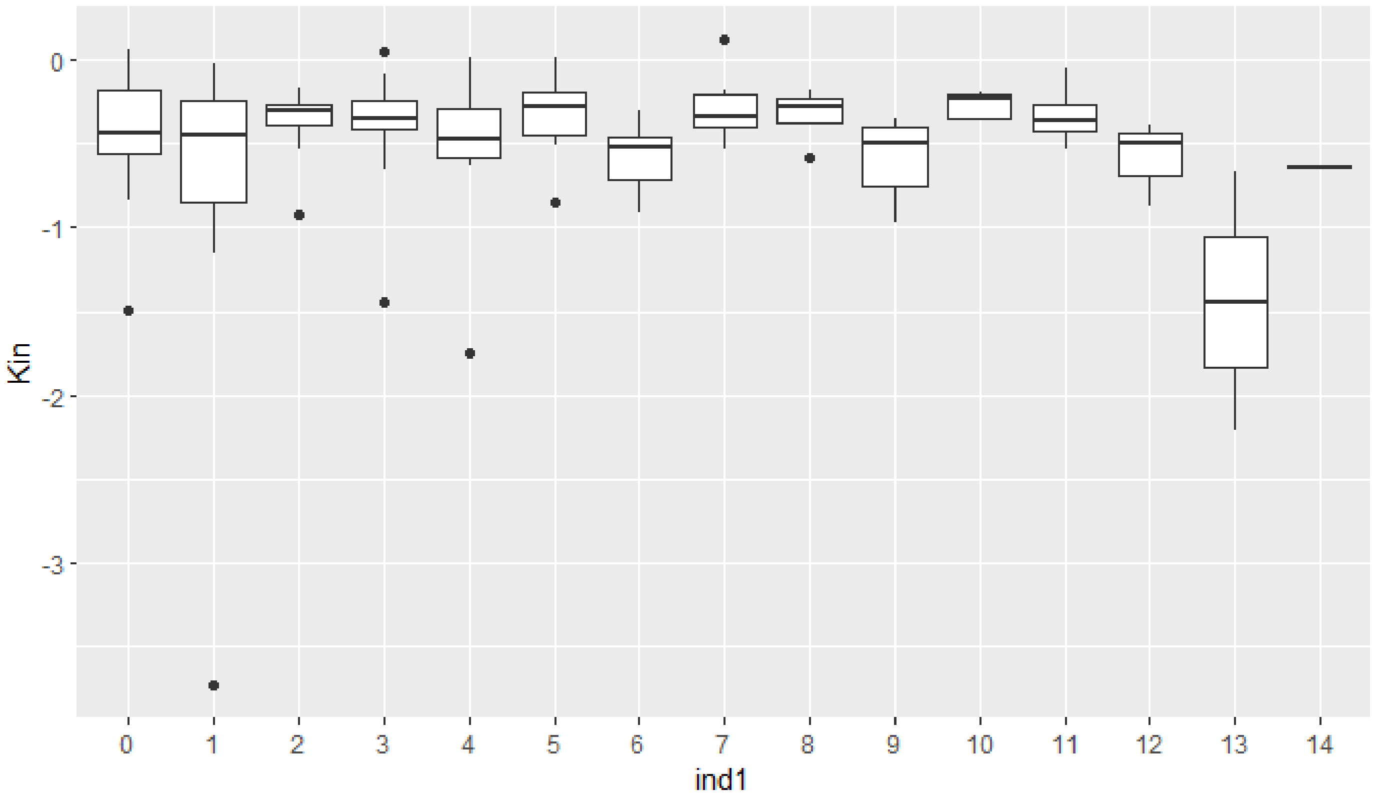

Figure S17: Estimates of kinship coefficients across pairwise comparisons from 16 *B. houstonensis* tadpoles sampled from three ponds in Lee County. We used IBSrelate to identify pairs of related individuals without requiring population allele frequencies and generated KING-robust kinship coefficient estimates for every pair. We observed negative estimates of kinship coefficients across pairs and concluded there was no relatedness among pairs, enabling inclusion of these samples for population genetic analyses.

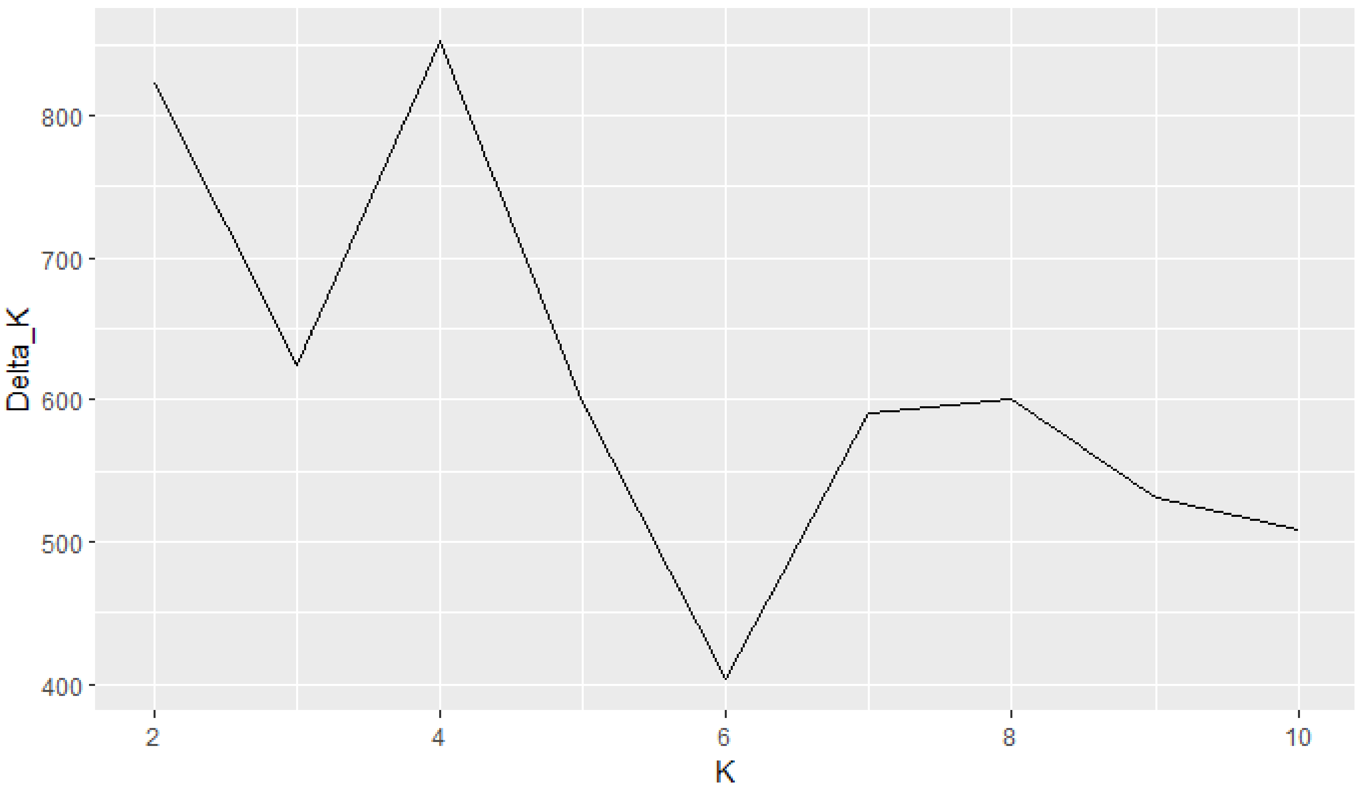

Figure S18: Most likely grouping level of *K*=4 detected during admixture analyses using 107,509 SNPs from 8 *Bufo* [=*Anaxyrus*] *americanus* and 48 *B*. *houstonensis* samples. We calculated Δ*K* from 10 independent runs in NgsAdmix, each of 20000 iterations.

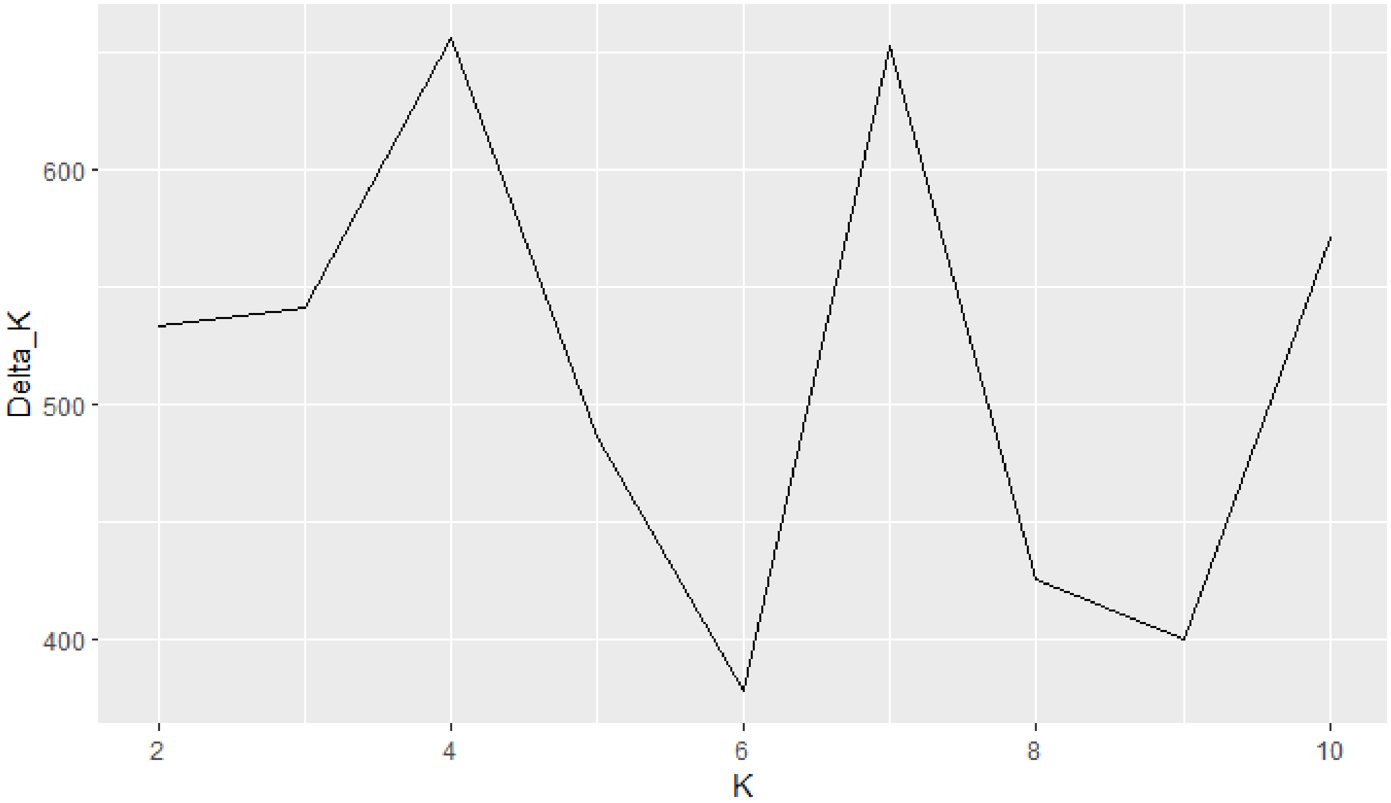

Figure S19: Most likely grouping level of *K*=4 detected during admixture analyses using 92,228 SNPs from 48 *Bufo* [=*Anaxyrus*] *houstonensis* samples. We calculated ΔK from 10 independent runs in NgsAdmix, each of 20000 iterations.
